# Dominant Role of Coexisting Ruthenium Nanoclusters Over Single Atoms to Enhance Alkaline Hydrogen Evolution Reaction

**DOI:** 10.1002/advs.202414012

**Published:** 2025-02-05

**Authors:** Jae‐Hoon Baek, Seong Hyeon Kweon, Hyuk‐Jun Noh, Do Hyung Kweon, Jeong‐Min Seo, Se Jung Lee, Sang Kyu Kwak, Jong‐Beom Baek

**Affiliations:** ^1^ School of Energy and Chemical Engineering/Center for Dimension‐Controllable Organic Frameworks Ulsan National Institute of Science and Technology (UNIST) Ulsan 44919 South Korea; ^2^ School of Energy and Chemical Engineering Ulsan National Institute of Science and Technology (UNIST) Ulsan 44919 Republic of Korea; ^3^ Department of Chemistry Burke Laboratory Dartmouth College Hanover New Hampshire 03755 USA; ^4^ Hydrogen Fuel Cell Research Center Korea Institute of Science and Technology (KIST) Seoul 02841 Republic of Korea; ^5^ Department of Chemical and Biological Engineering Korea University Seoul 02841 Republic of Korea

**Keywords:** alkaline hydrogen evolution, anion exchange membrane water electrolyzer, electrocatalysts, nanoclusters, single atoms

## Abstract

Developing efficient and cost‐effective electrocatalysts to replace expensive carbon‐supported platinum nanoparticles for the alkaline hydrogen evolution reaction remains an important challenge. Recently, an innovative catalyst, composed of ruthenium single atoms (Ru_1_) integrated with small Ru nanoclusters (Ru_NC_), has attracted considerable attention from the scientific community. However, because of its complexity, this catalyst remains a topic of some debate. Here, a method is reported of precisely controlling the ratios of Ru_1_ to Ru_NC_ on a nitrogenated carbon (NC)‐based porous organic framework to produce Ru/NC catalysts, by using different amounts (0, 5, 10 wt.%) of reducing agent. The Ru/NC–10 catalyst, formed with 10 wt.% reducing agent, delivered the best performance under alkaline conditions, indicating that Ru_NC_ played a significant role in actual alkaline hydrogen evolution reaction (HER). An anion exchange membrane water electrolyzer (AEMWE) system using the Ru/NC–10 catalyst required a significantly lower operating voltage (1.72 V) than the commercial Pt/C catalyst (1.95 V) to achieve 500 mA cm^−2^. Moreover, the system can be operated at 100 mA cm^−2^ without notable performance decay for over 180 h. Theoretical calculations supported these experimental findings that Ru_1_ contributed to the water dissociation process, while Ru_NC_ is more actively associated with the hydrogen recombination process.

## Graphical abstract

High‐performance ruthenium on nitrogenated carbon (Ru/NC) catalysts are synthesized with precisely controlled ratios of Ru single atoms (Ru_1_) and nanoclusters (Ru_NC_). Ru_1_ facilitated water dissociation, while Ru_NC_ enhanced hydrogen recombination. The Ru/NC–10 catalyst, formed with a 10% reducing agent, outperformed commercial Pt/C, demonstrating superior efficiency and durability in alkaline hydrogen evolution reaction (HER) and anion exchange membrane water electrolzyer (AEMWE) systems. A higher ratio of Ru_NC_ to Ru_1_ is crucial for improved water‐splitting performance.

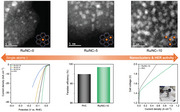

## 1 Introduction

The growing risks of climate change and energy security have increased the urgency of efforts to develop sustainable energy solutions. Hydrogen, as a promising alternative energy carrier to fossil fuels, has spurred the development of numerous production methods. Among them, water electrolysis has gained considerable attention because of its compatibility with renewable energy sources.^[^ ^1^ ^]^ This synergy is particularly important considering the intermittent nature of renewable energy sources, which accordingly suffer from variability and unpredictability.^[^ ^2^ ^]^ By driving water electrolysis with renewable energy, green hydrogen can be produced, providing a clean, storable, and transportable energy carrier that can help stabilize the energy supply.^[^ ^3^ ^]^ The most prevalent water electrolysis technologies are alkaline water electrolysis (AWE) and proton exchange membrane water electrolysis (PEMWE). While AWE is robust and cost‐effective, it has comparatively lower performance and efficiency than other methods. In contrast, PEMWE offers enhanced efficiency but comes with drawbacks, including expensive catalysts and system components, as well as harsh acidic operating conditions.^[^ ^4^ ^]^ To address these limitations, anion exchange membrane water electrolysis (AEMWE) has emerged as a promising alternative for large‐scale industrial hydrogen production. AEMWE combines the advantages of both AWE and PEMWE, providing a lower‐cost system with the potential for high efficiency.^[^ ^5^ ^]^ However, AEMWE still faces challenges, particularly in achieving the desired current density at a specific cell voltage, largely due to the slower hydrogen evolution reaction (HER) kinetics in alkaline media.

It is well established that the HER proceeds at a slower rate in alkaline conditions, with efficiency reduced by approximately two‐ to three‐fold compared to acidic conditions. In acidic environments, protons are readily available to drive the HER. In contrast, alkaline conditions hinder the reaction because an additional water dissociation step is required to generate protons.^[^ ^6^ ^]^ This results in markedly reduced efficiency and underscores the need for highly efficient and durable electrocatalysts specifically designed for alkaline HER. Platinum (Pt) and its alloys have long been the best catalyst for the HER due to their optimal hydrogen adsorption energy.^[^ ^7^ ^]^ However, its high cost and limited capacity for water dissociation significantly reduce overall performance in alkaline environments, where water dissociation is a critical step in the HER process.

To address these challenges, ruthenium (Ru)‐based catalysts have emerged as promising alternatives to Pt for alkaline HER because of their comparable hydrogen bonding energy, superior water dissociation ability, and lower cost.^[^ ^8^ ^]^ Recent studies have highlighted the critical importance of synergistic interactions between multiple active sites within a single catalyst structure, as these interactions facilitate multistep reactions, effectively reducing kinetic barriers and improving catalytic performance.^[^ ^9^ ^]^ Among the various approaches, the combination of Ru single atoms (Ru_1_) and Ru nanoclusters (Ru_NC_) has attracted significant attention. The coupling of Ru_1_ with Ru_NC_ enhances electronic interactions, accelerates water dissociation, and optimizes hydrogen adsorption and desorption.^[^ ^9b^ ^]^ Despite these advancements, previous studies have largely focused on size modulation of Ru_NC_ and Ru nanoparticles (Ru_NP_) and/or have been restricted to neutral pH conditions, leaving critical gaps between understanding the distinct roles and optimizing the ratio of Ru_1_ and Ru_NC_ under alkaline HER conditions. Furthermore, inconsistencies in experimental parameters, such as heat‐treatment protocols and Ru contents, have led to ambiguous conclusions.^[^ ^10^ ^]^ Bridging these knowledge gaps is essential for fully realizing the potential of Ru‐based catalysts to achieve efficient alkaline HER.

Here, we report the fabrication of electrocatalysts with Ru_1_ and Ru_NC_ on nitrogenated carbon (NC)‐based porous organic frameworks (Ru/NC). The ratios of Ru_1_ to Ru_NC_ in the Ru/NC catalysts were precisely controlled by carefully controlling the quantity of reducing agent while maintaining consistent Ru contents during heat‐treatment processes. Among Ru/NC catalysts formed with different amounts of reducing agent (0, 5, or 10 wt.%), the Ru/NC–10 catalyst (10 wt.% reducing agent) demonstrated an overpotential of 15.8 mV at 10 mA cm^−2^ in 1.0 м aq. KOH solution. In a two‐electrode system, it achieved a Faradaic efficiency of 98.5%, outperforming a commercial Pt/C catalyst (95.0%). Additionally, a full anion exchange membrane water electrolyzer (AEMWE) device required only 1.72 V to reach 500 mA cm^−2^ and exhibited excellent durability while operating at 100 mA cm^−2^ for 180 h. These results confirmed that the presence of Ru_NC_ played a more significant role under the coexistence condition with Ru_1_.

## 2 Results and Discussion

**Figure 1:**
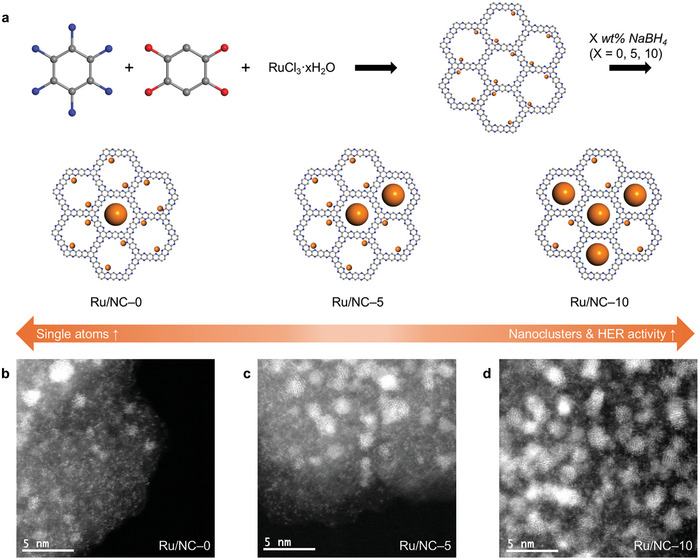
a) Schematic representation of the synthesis of Ru/NC–0, Ru/NC–5, and Ru/NC–10 catalysts formed with different amounts of sodium borohydride as a reducing agent (blue: nitrogen, gray: carbon, red: oxygen, orange: ruthenium). HAADF‐STEM images. b) Ru/NC–0; c) Ru/NC–5; d) Ru/NC–10.

The ratios of Ru_1_ to ultra‐small Ru_NC_ on the hydrophenazine‐linked fused aromatic network (HP‐FAN) could be precisely controlled by adjusting the amount of reducing agent (sodium borohydride, NaBH_4_). The goal was to achieve a uniform dispersion of Ru_1_ and Ru_NC_ on the NC support to yield Ru/NC–0 (0 wt.% NaBH_4_), Ru/NC–5 (5 wt.% NaBH_4_), and Ru/NC–10 (10 wt.% NaBH_4_) catalysts. HP‐FAN, a well‐defined nitrogenated carbon (NC)‐based porous organic framework, was selected as the NC support because of its uniformly distributed pores containing 12 nitrogen (N) atoms in each pore.^[^ ^11^ ^]^ As a result, the NC support could provide a high specific surface area, multiple anchoring N sites, and a conductive nature due to its π‐conjugated fused aromatic network structure. Ru single atoms were embedded in the HP‐FAN layers by in situ synthesis via a polycondensation reaction between hexaaminobenzene (HAB) trihydrochloride^[^ ^12^ ^]^ and 2,5‐dihydroxy‐1,4‐benzoquinone (DHBQ) in the presence of ruthenium(III) chloride hydrate (RuCl_3_·xH_2_O) as the Ru precursor. The HP‐FAN structure was confirmed with Fourier‐transform infrared (FTIR) data and the mass spectrum of the HAB and DHBQ monomers (Figure S1, Supporting Information).^[^ ^11^, ^12^ ^]^ Additionally, the structures of Ru‐loaded Ru/NC–X samples (where X represents the weight percent of the reducing agent) were further validated using FTIR data. The bands observed at 1252, 1446, 1623, and 3423 cm⁻¹ correspond to the C─N stretching, C═C stretching, C═N stretching, and the amine (C─NH─C) bond associated with the HP linkage, respectively (Figure S2, Supporting Information).^[^ ^11^ ^]^ The Brunauer–Emmett–Teller (BET) surface area analysis further confirmed its extensive surface area (Figure S3, Supporting Information). By carefully adjusting the amount of reducing agent and employing subsequent heat‐treatment, Ru/NC–X catalysts could be synthesized with distinct ratios of Ru_1_ and Ru_NC_ (**Figure** **1**a).

High‐angle annular dark‐field scanning transmission electron microscopy (HAADF‐STEM) analysis revealed that Ru single atoms were uniformly dispersed in the pre‐annealed (as‐prepared) sample with no reducing agent (Ru/NC–0‐b, before heat‐treatment). As the amount of reducing agent increased from 5 (Ru/NC‐5‐b) to 10 wt.% (Ru/NC‐10‐b), Ru single atoms began to form Ru_NC_ (Figure S4, Supporting Information). After heat‐treatment at 700 °C, Ru/NC–10 exhibited the highest concentration of Ru_NC_, suggesting that the presence of larger nanoclusters before heat‐treatment played a crucial role in seeding the further formation of Ru_NC_ during heat‐treatment.^[^ ^13^ ^]^ Energy‐dispersive X‐ray spectroscopy (EDS) elemental mapping of the Ru/NC–X (X = 0, 5, 10) catalysts confirmed the presence of Ru, N, and C elements (Figures S5–S7, Supporting Information). Ru_NC_ is uniformly distributed throughout the sample with an average diameter of ≈1.6 nm (Figure S8, Supporting Information). The crystal structure of the Ru/NC–X was analyzed using high‐power X‐ray diffraction (HP‐XRD) (Figure S9, Supporting Information). The diffraction peak at 25.4° corresponded to the (002) plane of the NC support, while peaks at 38.5, 42.2, 44.1, and 58.4° were respectively assignable to the (100), (002), (101), and (102) planes of hexagonal Ru nanocrystals (PDF no. 06–0633). Thermogravimetric analysis (TGA) under air conditions revealed that the Ru loading in Ru/NC–X samples was ≈13 wt.%, consistent with elemental analysis results (Figure S10 and Table S1, Supporting Information).^[^ ^14^ ^]^

**Figure 2:**
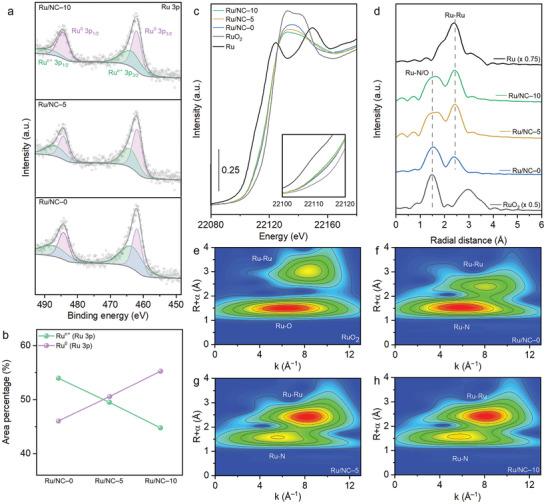
a) High‐resolution XPS spectra of Ru 3p for Ru/NC–0, Ru/NC–5, and Ru/NC–10. b) XPS peak area ratios for Ru‐N and Ru‐Ru in Ru/NC–0, Ru/NC–5, and Ru/NC–10. c) Ru K‐edge XANES spectra for Ru/NC–0, Ru/NC–5, Ru/NC–10, RuO_2_, and Ru foil (inset: magnified pre‐edge profiles of Ru K‐edge XANES), d) Fourier‐transformed Ru K‐edge EXAFS spectra for Ru/NC–0, Ru/NC–5, Ru/NC–10, RuO_2_, and Ru foil. e‐h) Wavelet transforms of the k^3^‐weighted EXAFS spectra for Ru/NC–0, Ru/NC–5, Ru/NC–10, and RuO_2_.

Powder X‐ray spectroscopy was used to elucidate the coexistence of Ru_1_ and Ru_NC_ within the Ru/NC–X samples (**Figure** **2**). X‐ray photoelectron spectroscopy (XPS) provided detailed information on the Ru 3p states before and after heat‐treatment. The XPS spectra revealed two distinct peaks, corresponding to metallic Ru (Ru^0^) at 462.0 eV and Ru_1_ ion (Ru^n+^) at 464.3 eV (Figure 2a).^[^ ^15^ ^]^ The Ru/NC–X‐b (before heat‐treatment) samples exhibited a higher percentage of Ru^n+^ peaks (Figure S11, Supporting Information). Conversely, the Ru/NC–X (after heat‐treatment) samples showed a larger ratio of peak areas corresponding to Ru_NC_ (Figure 2b; Table S2, Supporting Information).^[^ ^10c^ ^]^

High‐resolution N 1s XPS spectra identified a peak at 398.7 eV which was attributed to the Ru─N bond in samples both before (Ru/NC–X‐b) and after heat‐treatment (Ru/NC–X). The Ru 3p XPS results indicated that the area of the Ru‐N peak decreased with increasing amounts of reducing agent (Figure S12, Supporting Information).^[^ ^16^ ^]^ Despite the general tendency of Ru_1_ to agglomerate into Ru_NC_ on the NC support, due to their high surface energy,^[^ ^17^ ^]^ the nitrogen‐rich HP‐FAN support effectively facilitated the uniform distribution of Ru_1_ by forming Ru‐N bonds, as confirmed by HAADF‐STEM and XPS analyses (Figures S4 and S11, Supporting Information). The heat‐treatment induced the thermal reduction of atomic Ru ion into crystalline Ru_NC_, which was stably sequestered on the NC support.^[^ ^13^ ^]^

To gain further insight into the structural morphology of Ru_1_ and Ru_NC_, X‐ray absorption near‐edge structure (XANES) and extended X‐ray absorption fine structure (EXAFS) spectra were measured at the Ru K‐edge (Figure 2c,d; Figures S13 and S14, Supporting Information). XANES analysis confirmed that the absorption edges of all Ru/NC–X and Ru/NC–X‐b samples were positioned between those of the Ru foil (Ru^0^) and RuO_2_ (Ru^4+^) (Figure 2c; Figures S15a,b, Supporting Information).^[^ ^18^ ^]^ This result indicated that the Ru species in Ru/NC–X had positive valence states on average, suggesting the presence of both Ru_1_ and Ru_NC_. In particular, the absorption edge of the Ru/NC–10 samples showed the most negative shift, indicating a higher prevalence of metallic Ru states compared to the other samples (Figure 2c, inset).^[^ ^19^ ^]^ This result was evidence of the highest population of Ru_NC_ among Ru/NC–X samples. Next, the Ru bonding morphology of the Ru/NC–X and Ru/NC–X‐b samples was investigated using EXAFS analysis (Figure 2d; Figure S15c, Supporting Information). The pre‐annealed samples exhibited a peak at ≈1.5 Å, corresponding to Ru─N bonding, which was more intense than the peak at ≈2.4 Å, indicative of metallic Ru.^[^ ^20^ ^]^ This result suggested that most of the Ru atoms were single atomic state in Ru/NC–X‐b. In contrast, after heat‐treatment, the Ru/NC–X samples showed more metallic Ru peaks with respect to the reducing agent, which was crucial to seeding the formation of more Ru_NC_. Wavelet transform (WT) analysis further confirmed the coexistence of Ru_1_ and Ru_NC_. The pre‐annealed samples predominantly exhibited Ru‐N bonding, whereas the post‐annealed samples showed coexisting Ru‐N and Ru‐Ru bonding (Figure 2e–h; Figure S16, Supporting Information). These collective results indicated that the heat‐treatment induced the thermal reduction of atomic Ru ions into Ru_NC_, which together served as highly efficient active sites for overall hydrogen generation.^[^ ^13^ ^]^

**Figure 3:**
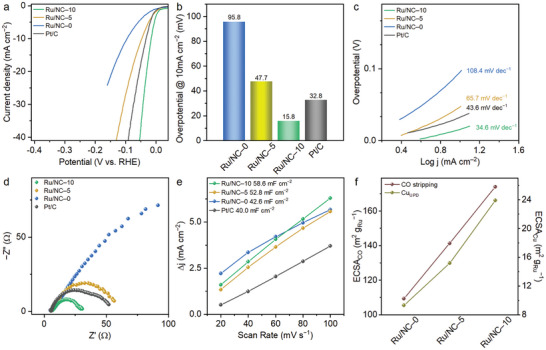
Electrochemical analyses of Ru/NC–0, Ru/NC–5, Ru/NC–10, and Pt/C in 1.0 m aq. KOH electrolyte. a) Polarization curves. b) Overpotential values at 10 mA cm^−2^. c) Tafel slopes. d) Charge resistance values. e) Double‐layer capacitances derived from CV curves. f) Electrochemical surface areas obtained from CO stripping and Cu_UPD_.

The HER activities of Ru/NC–X and the reference Pt/C catalysts were assessed in a 1.0 m aqueous potassium hydroxide (KOH) solution saturated with argon, and the results of the linear sweep voltammetry (LSV) measurements are shown (**Figure** **3**a). The Ru/NC–10 catalyst, with the highest Ru_NC_, exhibited the most effective HER activity, even surpassing the commercial Pt/C (20 wt.% Pt) catalyst. The Ru/NC–10 catalyst exhibited an overpotential of only 15.8 mV, which was lower than that of Ru/NC–0 (95.8 mV), Ru/NC–5 (47.7 mV), and Pt/C (32.8 mV) (Figure 3b; Table S4, Supporting Information). Additionally, its low Tafel slope indicated significant improvement in HER current density with increasing overpotential. The Tafel slope for the Ru/NC–10 catalyst was 34.6 mV dec^−1^, lower than Ru/NC–0 (108.4 mV dec^−1^), Ru/NC–5 (65.7 mV dec^−1^), and Pt/C (43.6 mV dec^−1^) (Figure 3c). Electrochemical impedance spectroscopy (EIS) analysis showed that the Ru/NC–10 catalyst had a lower charge transfer resistance compared to Pt/C at an overpotential of 25 mV (Figure 3d). To investigate the effect of the reducing agent, we synthesized an additional sample with a higher reducing agent concentration (Ru/NC–15). However, the catalytic activity was found to be saturated at the Ru/NC–10 levels (Figure S17, Supporting Information). The increased amount of reducing agent led to the growth of Ru nanoclusters into larger nanoparticles, which slightly decreased catalytic activity. The average particle size of Ru/NC–15 was measured to be 1.9 nm, compared to 1.6 nm for Ru/NC–10 (Figure S18, Supporting Information). Based on these results, the Ru/NC–10 catalyst was thought to have an optimal ratio and size of Ru_1_ and Ru_NC_, providing the most suitable electronic structure for HER performance.^[^ ^21^ ^]^

The high performance of the Ru/NC–10 catalyst could also be attributed to its high electrochemical specific area (ECSA), reflecting the number of active sites on the catalyst. The cyclic voltammetry (CV) curves measured at different scan rates revealed that the Ru/NC–10 catalyst had the largest double layer capacitance (C_dl_) (Figure 3e; Figure S19, Supporting Information).^[^ ^22^ ^]^ The number of electrochemically active sites was determined using two different methods: Cu‐underpotential deposition (Cu_UPD_) and CO stripping methods.^[^ ^8^ ^]^ With both methods, the Ru/NC–10 catalyst recorded the highest values compared to other samples (Figure 3f; Figures S20 and S21, Supporting Information). Collectively, these results indicated that the Ru/NC–10 catalyst had the highest number of active sites, and therefore exhibited superior HER performance.

To elucidate the roles of the Ru_1_ and Ru_NC_, poisoning tests were performed in a 1.0 m aq. KOH solution, containing 10 mM each of ethylenediaminetetraacetic acid disodium (EDTA) and potassium thiocyanate (KSCN).^[^ ^23^ ^]^ A significant reduction in HER activity was observed in the KSCN poisoning test, which bound to both Ru_1_ and Ru_NC_, while a lesser reduction was observed in the EDTA poisoning test, which bound mainly to Ru_1_ (Figure S22a, Supporting Information).^[^ ^24^ ^]^ This indicated that both Ru_1_ and Ru_NC_ were important to the HER activity. In particular, the Tafel slope value (120.4 mV dec^−1^) obtained from the EDTA poisoning test confirmed that Ru_1_ affected the water dissociation process, which is an important step in the alkaline HER reaction (Figure S22b, Supporting Information).^[^ ^9b^ ^]^ To gain further insight into the distinct roles and synergistic effects of Ru_1_ and Ru_NC_ in the HER, catalysts containing only Ru_1_ (Ru_1_/NC, referred to be Ru/NC–0‐b) and only Ru_NC_ (Ru/C) were evaluated (Figure S23, Supporting Information). The results demonstrated that the Ru/C catalyst exhibited significantly higher activity compared to Ru₁/NC, even rivaling the performance of the commercial Pt/C catalyst. These findings highlight the pivotal role of the Ru_NC_ active site in effectively driving HER under alkaline conditions. The experiments demonstrated that the coexistence of Ru_1_ and Ru_NC_ systems was crucial for optimal HER performance, with Ru_NC_ playing a particularly dominant role in enhancing activity.

The long‐term stability of the Ru/NC–10 catalyst was further evaluated with LSV analysis after 10 000 cycles. This analysis showed only a slight increase in overpotential, of 1.1 mV, indicating robust stability (Figure S24, Supporting Information).^[^ ^25^ ^]^ After the stability test, HR‐TEM imaging confirmed that the Ru_NC_ was uniformly well‐dispersed and remained intact on the NC support (Figure S25, Supporting Information). Additionally, HP‐XRD patterns confirmed that the crystallinity of the Ru/NC–10 catalyst remained intact after the stability test (Figure S26, Supporting Information). XPS analysis further confirmed the stable retention of Ru_1_ sites and the nitrogen‐doped carbon structure, indicating no significant degradation in their chemical composition (Figure S27, Supporting Information). HAADF‐STEM elemental mapping images verified the consistent distribution of Ru and other elements on the NC support, reinforcing the structural integrity of the catalyst (Figure S28, Supporting Information). These results collectively demonstrate that the Ru/NC–10 catalyst retains its structure and chemical properties even after prolonged operation.

In the alkaline HER, the role of hydroxide ions (OH^−^) produced during water dissociation was significant. If the OH^−^ ions were strongly bound to the metallic Ru active sites, they could obstruct these sites.^[^ ^26^ ^]^ CO stripping was employed to assess the OH^−^ affinity of the sample, as the electrochemical oxidation of CO was initiated by the adsorption of active OH^−^. A negative shift in the CO oxidation potential generally indicates a strong OH^−^ affinity for the catalyst.^[^ ^27^ ^]^ The Ru/NC–10 samples exhibited the highest initial potential (0.39 V), indicating that Ru‐OH adsorption was the weakest compared to the other samples. Moreover, the difference in initial CO oxidation potentials highlighted significant variations in the electronic structures of the active sites across Ru/NC–0, Ru/NC–5, and Ru/NC–10, suggesting that the Ru/NC–10 catalyst had the optimal composition for HER (Figure S21, Supporting Information).

Density functional theory (DFT) calculations were performed to theoretically elucidate the superior alkaline HER activity of the catalysts with coexisting Ru_1_ and Ru_NC_. First, the catalyst structure (i.e., Ru_1_+Ru_NC_) of coexisting Ru_1_ and Ru_NC_ was constructed. For comparison, the structures of Ru_1_ and Ru_NC_ were also constructed, respectively. Based on the results of the experimental XPS and XAFS spectra analysis data (Figure S12 and Table S3, Supporting Information), the Ru─N functional group was considered for the nitrogen (N) sites bonded with the Ru_1_, and the pyridinic‐N and pyrrolic‐N functional groups were considered together for the N sites bonded with the Ru_NC_ (Figure S29, Supporting Information). Then, the water adsorption energy for each structure (i.e., Ru_1_, Ru_NC_, or Ru_1_+Ru_NC_, Figure S30, Supporting Information) was calculated to investigate the water dissociation in the alkaline HER. In the case of Ru_1_+Ru_NC_, two distinct active sites were separately considered. The first is where Ru_1_ serves as an active site (i.e., Ru_1_(Ru_1_+Ru_NC_)), and the second is where Ru_NC_ is the active site (i.e., Ru_NC_(Ru_1_+Ru_NC_)). The calculation results showed that the Ru_1_(Ru_1_+Ru_NC_) site had greater adsorption energy (i.e., −0.98 eV) compared to other active sites, indicating that the Ru_1_ site in the coexisting catalyst of Ru_1_ and Ru_NC_ has the strongest interaction with H_2_O (Figure S31, Supporting Information).

**Figure 4:**
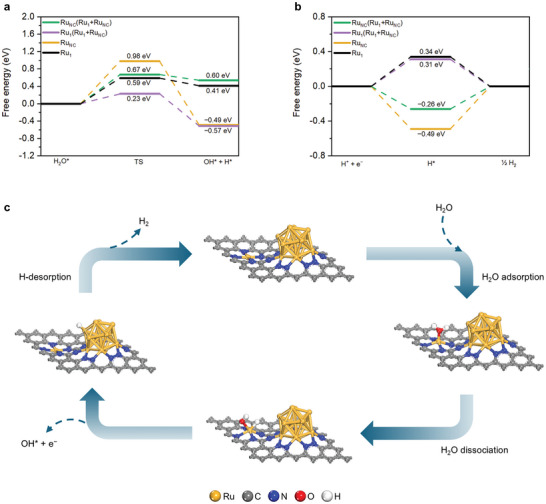
a) The calculated free energy diagrams of the water dissociation process on different active sites. b) The calculated free energy diagrams of H‐adsorption on different active sites. Note that the Ru single atom site, Ru nanocluster site, coexisting Ru single atom site, and coexisting Ru nanocluster site are denoted as Ru_1_, Ru_NC_, Ru_1_(Ru_1_+Ru_NC_) and Ru_NC_(Ru_1_+Ru_NC_), respectively. c) Schematic diagram of the synergistic effect mechanism among Ru_1_ site, and Ru_NC_ site in coexistent systems in the alkaline HER process.

Next, the free energy calculation in the water dissociation process was performed for each structure (Figure S32, Supporting Information). Notably, the activation barrier and water dissociation energy at the Ru_1_(Ru_1_+Ru_NC_) site were 0.23 and −0.57 eV, respectively, which were lower than those of the other active sites (**Figure** **4**a). This explains why the Ru_1_ and Ru_NC_ coexisting catalyst outperformed the other independent catalysts (i.e., Ru_1_ or Ru_NC_) for excellent HER, with Ru_1_ acting as the key water dissociation site.

Subsequently, we calculated the free energy of hydrogen adsorption to check the feasibility of hydrogen desorption during the alkaline HER reaction (Figure 4b). The free energies at the Ru_1_ and Ru_1_(Ru_1_+Ru_NC_) sites showed positive values of 0.34 and 0.31 eV, respectively, indicating that weakly bound hydrogen could desorb from the Ru_1_ site. On the other hand, the free energies at the Ru_NC_ and Ru_NC_(Ru_1_+Ru_NC_) sites showed negative values, and the latter was close to 0 eV (i.e., −0.26 eV). Thus, the Ru_NC_(Ru_1_+Ru_NC_) site could be considered the optimal for adsorption of hydrogen as a clear indication of hydrogen initially adsorbed on Ru_1_(Ru_1_+Ru_NC_) migrating to Ru_NC_(Ru_1_+Ru_NC_).^[^ ^9c^ ^]^ Furthermore, the OH^−^ adsorption strength at the Ru_NC_(Ru_1_+Ru_NC_) site was weaker (i.e., −0.41 eV) than those at other sites as shown by the OH^−^ adsorption energy (Figure S31, Supporting Information), consistent with previous data (Figure S21, Supporting Information). Therefore, the Ru_NC_ site in the coexisting catalyst is expected to play a dominant role in HER performance, as it not only facilitates OH^−^ desorption during water dissociation but also exhibits optimal hydrogen adsorption strength.

In conclusion, it is understood that the Ru_1_(Ru_1_+Ru_NC_) site plays a dominant role in the water dissociation process, and the Ru_NC_(Ru_1_+Ru_NC_) site plays a dominant role in the H‐recombination process (Figure 4c). The excellent HER catalytic performance of the coexisting catalyst is attributed to the synergistic effect of the Ru_1_ atom and Ru_NC_.

A full‐cell test was conducted in an overall water‐splitting system to investigate practical energy device applications. The two‐electrode device was fabricated with electrodes for oxygen (OER) and hydrogen evolution reactions (HER). Nickel foam (NF) served as the substrate for the electrodes, which were coated with catalysts using electrospray (Figure S33, Supporting Information). Ru/NC–10 and Pt/C coated on NF were evaluated as HER electrodes, while commercial iridium oxide (IrO_2_) coated on NF served as the OER electrode.

**Figure 5:**
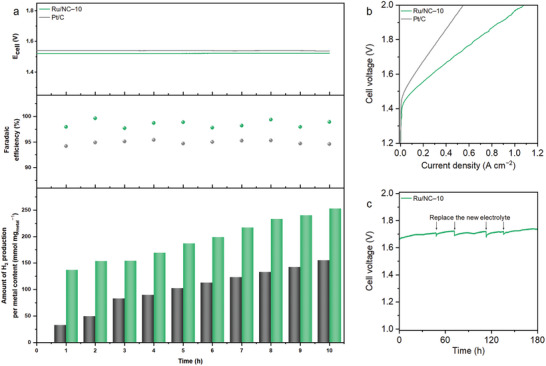
Overall water splitting system. a) Variation in cell potential at a constant current, Faradaic efficiency, and the amount of H_2_ production per metal content at a specific current of 5 mA. b) Polarization curves of AEMWE operated at 70 °C. c) Stability tests of AEMWE at a specific current density of 100 mA cm^−2^ at ambient conditions.

To accurately quantify hydrogen generation, systematic experiments were performed by connecting a closed water‐splitting cell (HER + OER) directly to a gas chromatography (GC) instrument.^[^ ^14^ ^]^ A constant current was applied to the system for 10 h, and hourly hydrogen production was measured. To achieve a current of 5 mA, the Ru/NC–10 catalyst required only 1.51 V, while the commercial Pt/C catalyst needed 1.54 V. The amount of hydrogen production of the Ru/NC–10 catalyst was considerably higher than that of the commercial Pt/C catalyst. In addition, the Faraday efficiency, an indicator of electron efficiency, demonstrated that the Ru/NC–10 catalyst exhibited 98.5%, which was higher than the commercial Pt/C catalyst (95.0%) (**Figure** **5**a).

To evaluate the practical application potential of the Ru/NC–10 catalyst, its activity, and stability were further assessed in an alkaline exchange membrane water electrolyzer (AEMWE) device (Figure S34, Supporting Information). In this setup, Ru/NC–10 served as the cathodic catalyst, while NiFe layered double hydroxides (LDH) were used as the anodic catalyst. The mass loading of Ru/NC–10 was maintained at 0.4 mg_Ru_ cm^−2^, and the benchmark was 20 wt.% Pt/C with 0.6 mg_Pt_ cm^−2^ was also tested for comparison. Despite the lower metal loading, the Ru/NC–10 catalyst exhibited higher current densities than commercial Pt/C at equivalent cell potentials. Specifically, Ru/NC–10 required only 1.72 V to achieve a current density of 500 mA cm^−2^, which was significantly lower than the 1.95 V required for Pt/C (Figure 5b). The chronopotentiometry curve, recorded at room temperature with a current density of 100 mA cm^−2^, demonstrated that the Ru/NC–10 catalyst maintained excellent stability for over 180 h (Figure 5c).

## 3 Conclusion

In conclusion, we demonstrated the synthesis of high‐performance ruthenium (Ru) on nitrogenated carbon support (Ru/NC) catalysts with a precisely controlled ratio of Ru single atoms (Ru_1_) to Ru nanoclusters (Ru_NC_) by regulating the reducing agent. Various characterization techniques revealed that, while the loading amount of Ru remained similar across samples, the ratios of Ru_1_ and Ru_NC_ could be precisely controlled. Both electrochemical measurements and DFT calculations indicated that Ru_1_ sites contributed to the water dissociation step, while the Ru_NC_ sites played a significant role in the hydrogen recombination step. The experimental results showed that increasing the proportion of Ru_NC_ enhanced the overall performance of the alkaline hydrogen evolution reaction. Specifically, the Ru/NC–10 catalyst required an overvoltage of only 15.8 mV to achieve a current density of 10 mA cm^−2^. It also demonstrated a Faraday efficiency of 98.5% in a two‐electrode system under full‐cell conditions. In the practical AEMWE system, the Ru/NC–10 catalyst achieved a current density of 500 mA cm^−2^ at a voltage of only 1.72 V, outperforming the commercial Pt/C catalyst (1.95 V). Moreover, the catalyst exhibited exceptional durability, maintaining stable performance at 100 mA cm^−2^ for 180 h, further supporting its practical applicability. Both experimental and theoretical results revealed that a higher ratio of Ru_NC_ to Ru_1_ played a more active role in alkaline HER performance. This study highlights the importance of strategically balancing Ru_1_ and Ru_NC_ ratios in catalyst design, providing valuable insights to optimize noble metal‐based catalysts to enhance efficiency and durability. This approach offers significant potential for developing high‐performance, cost‐effective solutions for practical hydrogen production.

## 4 Experimental Section

### Sample Preparation

RuCl_3_·xH_2_O (45 mg) was dissolved in N‐methyl‐2‐pyrrolidone (NMP) (3 mL) and the solution was placed in an ice bath within a one‐necked round bottom flask while stirring for 30 min. Hexaaminobenzene (HAB; 132 mg) trihydrochloride and 2,5‐dihydroxy‐1,4‐benzoquinone (DHBQ; 100 mg) were added to the mixture under an argon atmosphere. The ice bath was then replaced with an oil bath, and the temperature gradually increased to 175 °C, where it was maintained for 16 h. Upon the completion of the reaction, the mixture was cooled to 80 °C, and a NaBH_4_ solution (2.4 mL) of varying concentrations (X = 0, 5, 10 wt.%) was slowly added under vigorous stirring. After the addition, the temperature was raised back to 175 °C and maintained for an additional 4 h. The reaction mixture was then cooled to room temperature, and the resulting black powder was collected by suction filtration using a polytetrafluoroethylene (PTFE) membrane. The black compound was subjected to Soxhlet extraction with acetone, ethanol, and methanol for 1 day, 2 days, and 1 day, respectively, and subsequently freeze‐dried at −120 °C under reduced pressure (0.005 mmHg). Finally, the product was annealed at 700 °C for 2 h under an argon atmosphere. The sample prepared prior to annealing was denoted as Ru/NC–X‐b, while the sample after annealing was referred to as Ru/NC–X. Specifically, the Ru_1_/NC sample corresponds to Ru/NC–0‐b.

### Characterizations

FTIR spectra were obtained using a Spectrum 100 (Perkin‐Elmer, USA) with KBr pellets. Mass spectra were recorded on an AccuTOF 4G+ DART. The low‐pressure nitrogen adsorption‐desorption isotherms were measured using a BELSORP‐max at 77 K over a relative pressure range of *P/P₀* = 10^−6^ to 0.99. Before the isotherm measurements, the samples were degassed under a high vacuum at 120 °C for 24 h. The Brunauer–Emmett–Teller (BET) model was utilized to determine the surface area, with the analysis conducted in the relative pressure range of 0.02 to 0.08 *P/P₀* for all samples. Elemental analysis (EA) was performed using a Thermo Scientific Flash 2000 analyzer. High‐resolution transmission electron microscopy (HR‐TEM) was conducted on a JEOL JEM‐2100F equipped with a field emission gun operated at 200 keV. Thermogravimetric analysis (TGA) was performed at a heating rate of 10 °C min^−1^ in air using a thermogravimetric analyzer (Q200, TA Instruments, USA). X‐ray diffraction (XRD) patterns were recorded on a high‐power X‐ray diffractometer (D/MAX 2500 V/PC, Rigaku, Japan) using Cu‐Kα radiation (35 kV, 20 mA, λ = 1.5418 Å). X‐ray photoelectron spectroscopy (XPS) was carried out using a K‐alpha spectrometer (Thermo Fisher Scientific, UK). Elemental analyses were performed on a Flash 2000 Analyzer. X‐ray absorption spectroscopy (XAS) was recorded on the 6D UNIST‐PAL beamline at the Pohang Light Source in Korea.

### Electrochemical Measurements

Electrochemical measurements were conducted using an electrochemical workstation (Ivium, Netherlands) with a standard three‐electrode setup. A graphite rod served as the counter electrode, and an Ag/AgCl (saturated KCl) electrode was used as the reference electrode. All potentials were referenced to the reversible hydrogen electrode (RHE). For the catalyst preparation, 5 mg of each catalyst was dispersed in 980 µL of isopropyl alcohol along with 20 µL of Nafion solution (5 wt.% in a mixture of lower aliphatic alcohol and water, Aldrich Chemical Inc.). The mixture was sonicated in an ice bath for 30 min and then stirred overnight at room temperature to produce a uniform catalyst ink. This ink was drop‐cast onto a rotating 4 mm diameter ring‐disk electrode (RRDE) to form a thin film for electrochemical testing. The catalyst ink loading amount was 30 µL. Linear sweep voltammetry (LSV) was performed in a 1.0 m aq. KOH solution at a scan rate of 5 mV s^−1^ and a rotation speed of 1600 rpm at room temperature. The solution resistance (Rr) in the 1.0 m aq. KOH solution was measured to be 6 Ω using Nyquist plots. An Ohmic drop (iR) correction was applied to all polarization curves. Electrochemical impedance spectroscopy (EIS) measurements were conducted by applying an AC amplitude of 10 mV over a frequency range of 0.1 to 10⁵ Hz against the Ag/AgCl reference electrode. Capacitance measurements were performed in a no faradaic potential region at scan rates of 20, 40, 60, 80, and 100 mV s^−1^. The correlation between the current produced in the electrochemical cell and the cathode's electrode potential represented using a Tafel plot, derived from the Tafel equation: η = *b* log *j* + *a*, where η is the overpotential value, *b* is the Tafel slope and *a* is a constant. The Tafel slope (*b*) was determined by analyzing the linear region of the steady‐state polarization curve in the η versus log *j* plot. Underpotential deposition (UPD) of copper was conducted in a 0.5 m aq. H₂SO₄ solution. Before conducting Cu stripping experiments, which were done in the absence and presence of 5 mM CuSO₄ at a scan rate of 10 mV s^−1^, a UPD layer was formed by polarizing the electrode at 0.230 V versus RHE for 100s. CO stripping experiments were conducted in a 1.0 m aq. KOH solution. The working electrode was initially held at 0.05 V versus RHE in a 1.0 m aq. KOH solution while a saturated CO/Ar gas mixture was bubbled through the solution for 10 min to poison the electrode. Following this, the electrolyte was purged with Ar for 10 min without applying any potential. Cyclic voltammetry (CV) was then performed from 0.05 to 1.0 V versus RHE at a scan rate of 10 mV s^−1^. The poisoning experiment was conducted in an electrolyte consisting of 10 mM KSCN and 10 mM EDTA in a 1.0 m aq. KOH solution. LSV was performed at a scan rate of 5 mV s^−1^.

### Computational Details

All density functional theory (DFT) calculations were performed using the Dmol^3^ program.^[^ ^28^ ^]^ The generalized gradient approximation (GGA) Perdew–Burke–Ernzerhof (PBE) exchange–correlation functional^[^ ^29^ ^]^ was used, and the core electrons were all treated as electrons with relativistic effects. The effective core potential was utilized for core treatment with a basis set of double numerical plus polarization (DNP) 4.4 level. Spin‐polarized calculations were employed, and a van der Waals correction was adopted by using the Grimme.^[^ ^30^ ^]^ The convergence criterion for self‐consistent calculation was set to 1 × 10^−6^ Ha. The convergence criteria for geometry optimization were set as 1 × 10^−5^ Ha, 0.002 Ha Å^−1^, and 0.005 Å for the maximum energy change, maximum force, and maximum displacement, respectively. To calculate the transition state in the reaction mechanism, the electron exchange‐correlation energy was calculated with the GGA and PBE functional. Complete single linear synchronous transit (LST) and quadratic synchronous transit (QST) methods were used.^[^ ^31^ ^]^ The root mean square (RMS) convergence force on the atoms was set to be 0.002 Ha Å^−1^.

The adsorption energies (i.e., ∆E_Adsorption_) were calculated according to the following equation:

(1) $$\Delta E_{\mathrm{Adsorption}}=E_{\mathrm{total}}\;-\left(E_{\mathrm{adsorbate}}+E_{\mathrm{surface}}\right)$$

where *E*_total_ represents the total energy of the surface with adsorption, and *E*_adsorbate_ and *E*_surface_ represent the adsorbates and surface, respectively. The H‐adsorption free energy (i.e., ∆G_H*_) was calculated according to the following equation:

(2) $$\Delta G_{H^{*}}=\Delta E_{\mathrm{Adsorption}}+\Delta \mathrm{ZPE}-T\Delta S$$

where ∆ZPE, *T*, and ∆*S* represent the change in the zero‐point energy, absolute temperature, and entropy change of hydrogen adsorption at room temperature, respectively. The ∆ZPE‐*T*∆*S* value was obtained according to Tiwari et al.^[^ ^32^ ^]^

The bulk structure of graphene was constructed using a primitive cell of graphite, containing two carbon atoms. Then, a graphene surface slab was modified to 6 × 6 × 1 supercells, which was enough to contain a Ru_13_ nanocluster. Moreover, the nitrogen functional groups doped on the graphene were considered, based on the experimental results (Figure S12 and Table S3, Supporting Information). In the surface model with a Ru single atom (i.e., Ru_1_), the Ru‐N4 functional group was considered, and in the surface containing Ru nanocluster (i.e., Ru_NC_), the pyridinic and pyrrolic nitrogen functional groups were considered together. In addition, the surface containing both a Ru_1_ and a Ru_NC_ was modeled based on the nitrogen functional groups contained on the Ru_1_ and Ru_NC_ surfaces (Figure S29, Supporting Information). Also, a vacuum region greater than 30 Å was introduced in the z‐direction in each Ru_1_, Ru_NC_, and Ru_1_+Ru_NC_ surface, respectively.

### Overall Water Splitting Measurement

The catalyst ink was prepared by dispersing 30 mg of catalyst in a mixture of water and isopropyl alcohol (IPA) at a 1:3 volume ratio, with a total volume of 15 mL. Nafion solution was added to achieve an ionomer‐to‐carbon (I/C) ratio of 0.7. The mixture was ultrasonicated for 1 h in an ice bath to ensure a homogeneous suspension. The prepared ink was then deposited onto nickel foam (NF) using an electrospray method. Specifically, the catalyst ink was loaded into a plastic syringe equipped with a 30‐gauge stainless steel hypodermic needle, which was connected to a high‐voltage power supply (ESN‐HV30). A voltage of ≈5.0 kV was applied between the needle and the NF substrate, positioned 8.5 cm apart. The ink was dispensed at a controlled flow rate of 75 µL min⁻¹ using a syringe pump (KD Scientific Model 220). The Fumasep FAA‐3‐PK‐130 membrane was pre‐treated by incubating it in a 1.0 m aq. KOH solution for 24 h, followed by thorough washing. Ru/NC–10 and Pt/C were used as cathode catalysts. The geometric area of the anion exchange membrane (AEM) electrolyzer was 1 cm^2^. The cathode and anode were sandwiched with the membrane to form the AEM electrolyzer, with catalyst loadings of 0.4 mg_Ru_ cm⁻^2^ for Ru/NC–10 and 0.6 mg_Pt_ cm⁻^2^ for 20 wt.% Pt/C. The AEM electrolyzer was operated at 70 °C with a 1.0 m aq. KOH solution flowing at a rate of 75 mL min⁻¹.

## Conflict of Interest

The authors declare no conflict of interest.

## Supporting information



Supporting Information

## Data Availability

The data that support the findings of this study are available from the corresponding author upon reasonable request.
