# Supplementary material for: Dominant Role of Coexisting Ruthenium Nanoclusters Over Single Atoms to Enhance Alkaline Hydrogen Evolution Reaction
Source: Adv Sci (Weinh). 2025 Feb 5;12(12):2414012. doi: 10.1002/advs.202414012 (PMC11948018; doi:10.1002/advs.202414012)
Supplement: Supplementary file 1 — Supporting Information [file ADVS-12-2414012-s001.docx]

Supporting Information

**Dominant Role of Coexisting Ruthenium Nanoclusters over Single Atoms to Enhance Alkaline Hydrogen Evolution Reaction**

*Jae-Hoon Baek, Seong Hyeon Kweon, Hyuk-Jun Noh, Do Hyung Kweon, Jeong-Min Seo, Se Jung Lee, Sang Kyu Kwak*, and Jong-Beom Baek**

**Table of Contents**

**Figures S1-S34**

**Tables S1-S4**

**References**


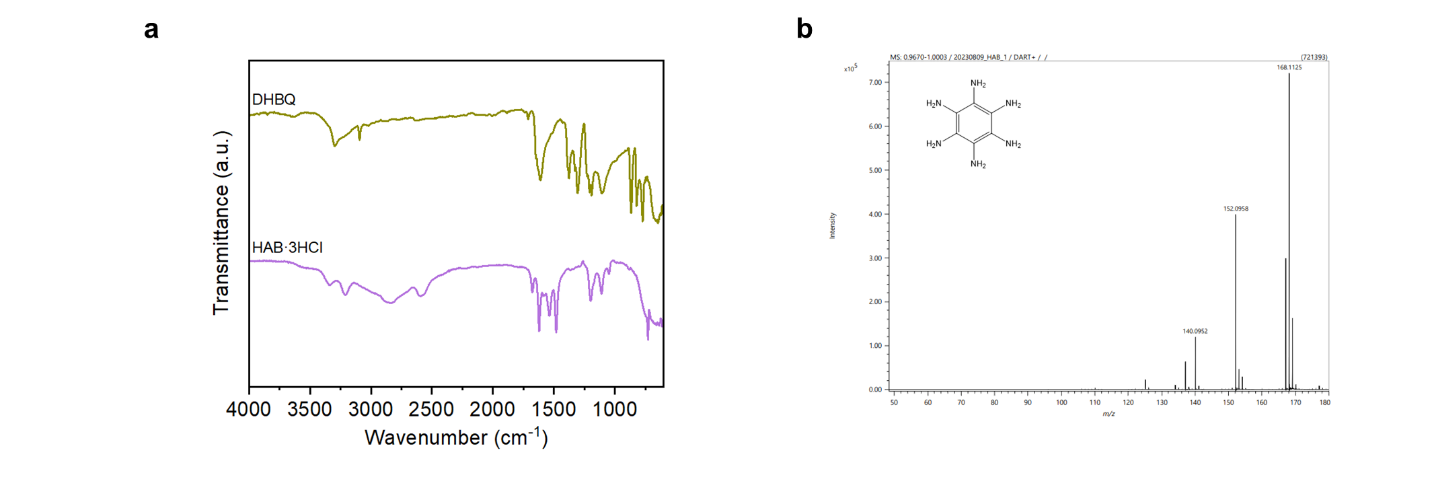


**Figure S1.** a) FTIR spectra of HAB and DHBQ monomers. b) Mass spectrum of HAB.


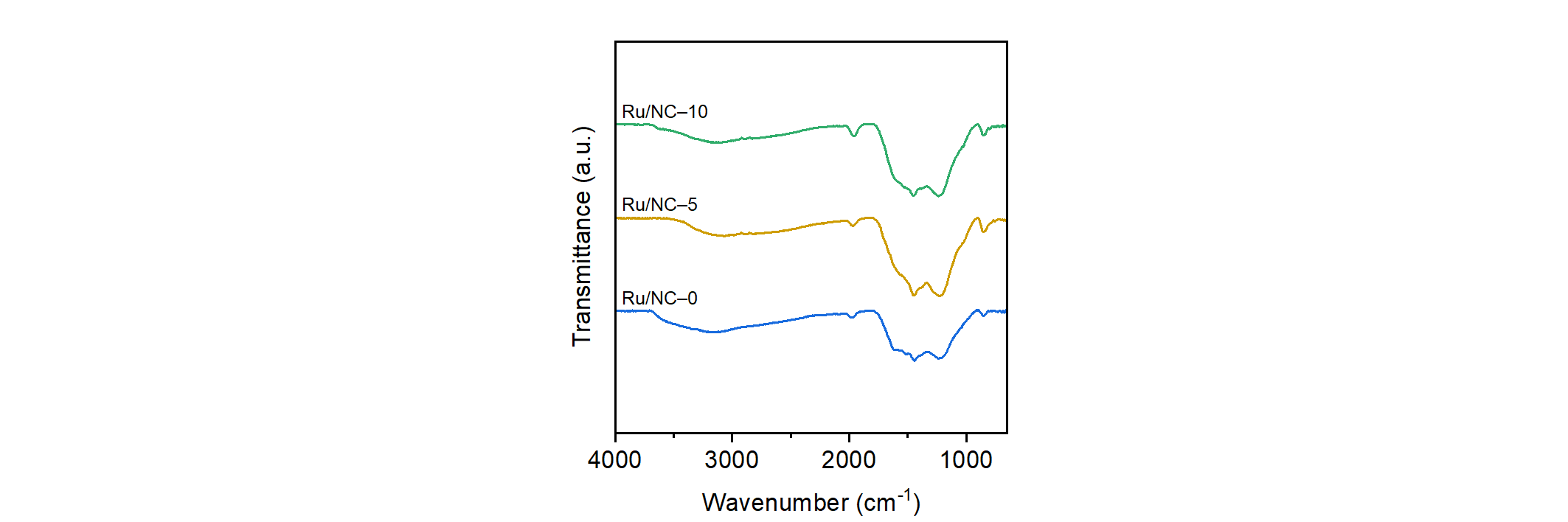
**Figure S2.** FTIR spectra of Ru/NC–0, Ru/NC–5, and Ru/NC–10.
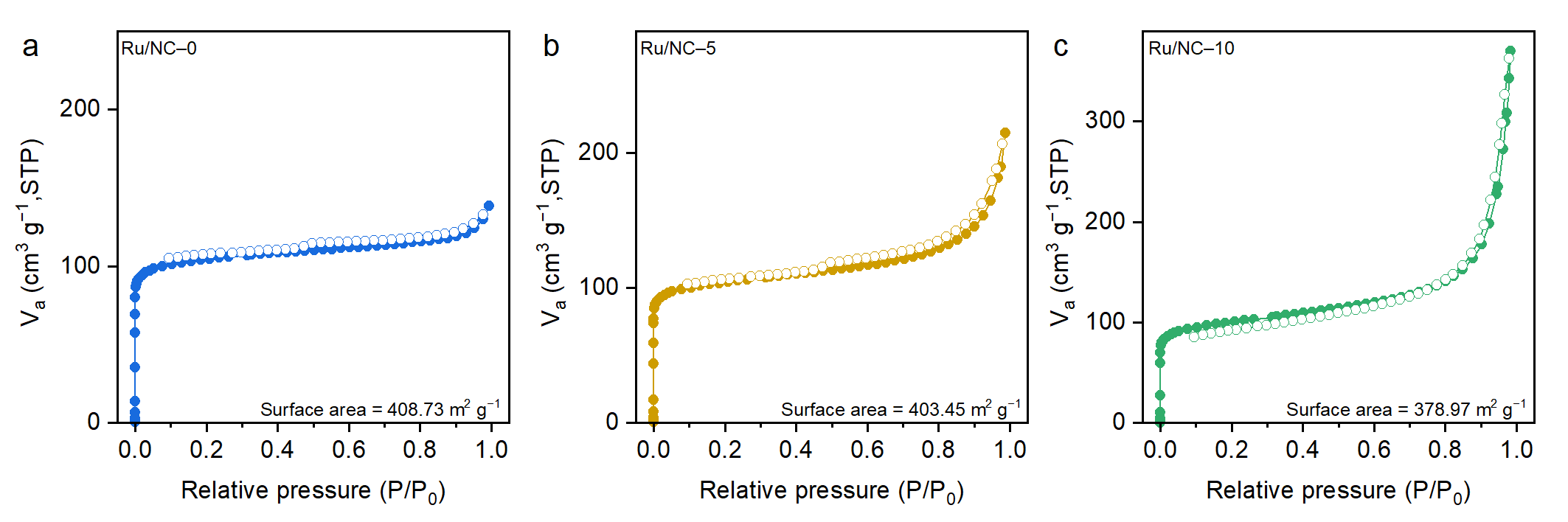


**Figure S3.** N_2_ sorption isotherms measured at 77 K. Filled circles: adsorption; open circles: desorption. a) Ru/NC–0; b) Ru/NC–5; c) Ru/NC–10.


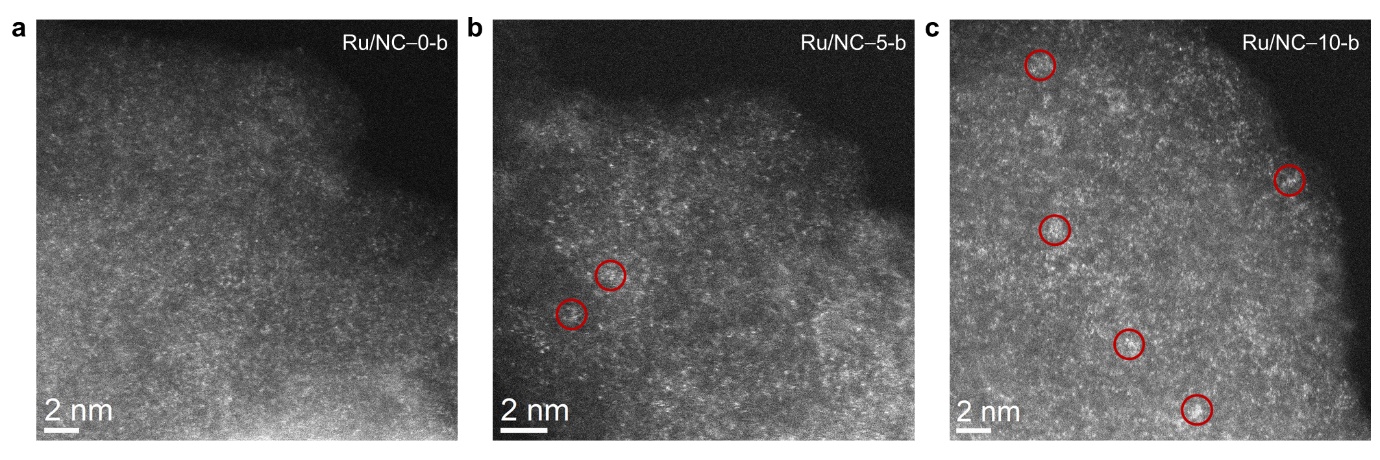


**Figure S4.** Atomic-resolution HAADF-STEM images. Red circles indicate Ru nanoclusters. a) Ru/NC–0-b; b) Ru/NC–5-b; c) Ru/NC–10-b.


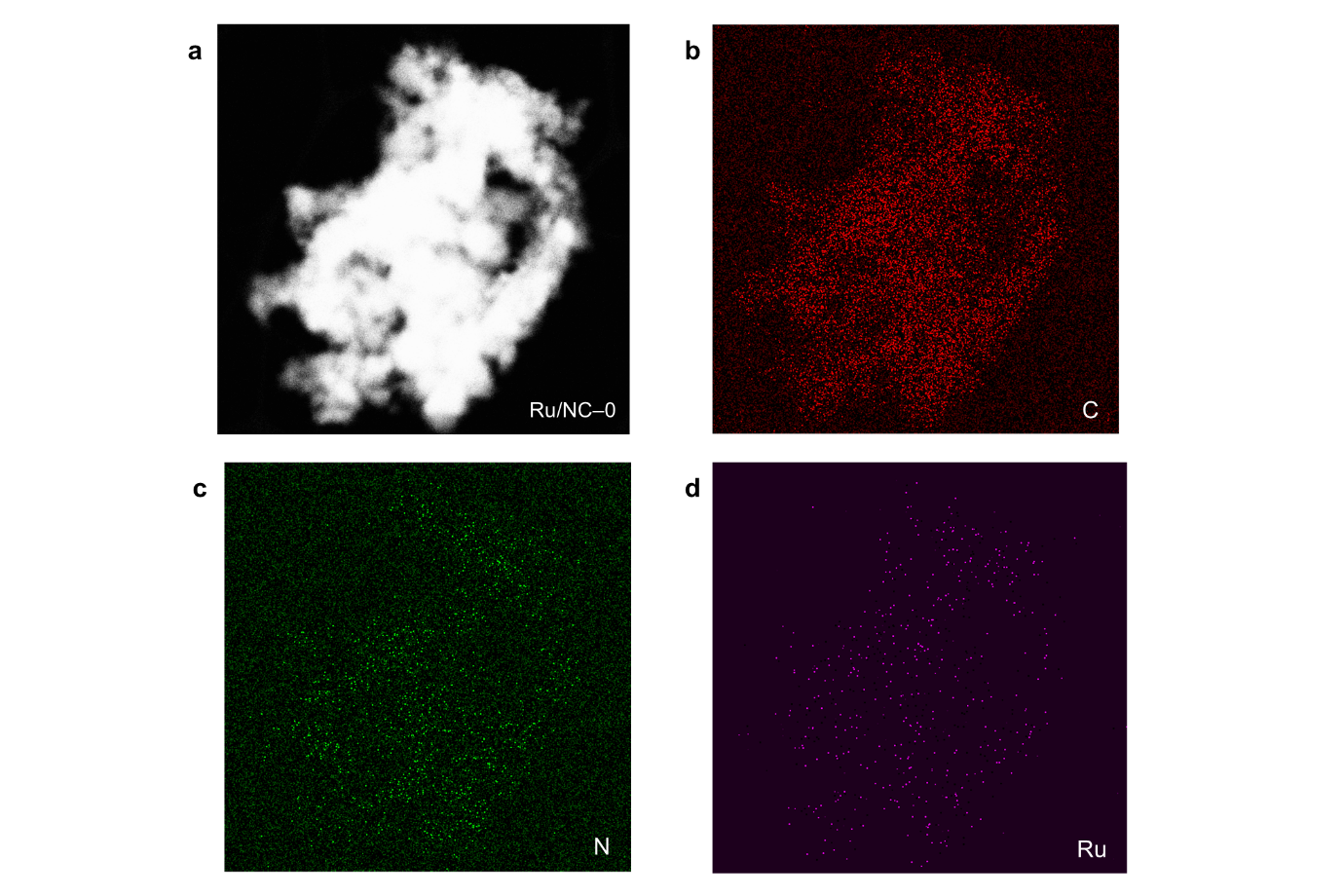


**Figure S5.** a) HAADF-STEM image of the Ru/NC–0. Corresponding EDS elementary mapping images. b) C; c) N; d) Ru.


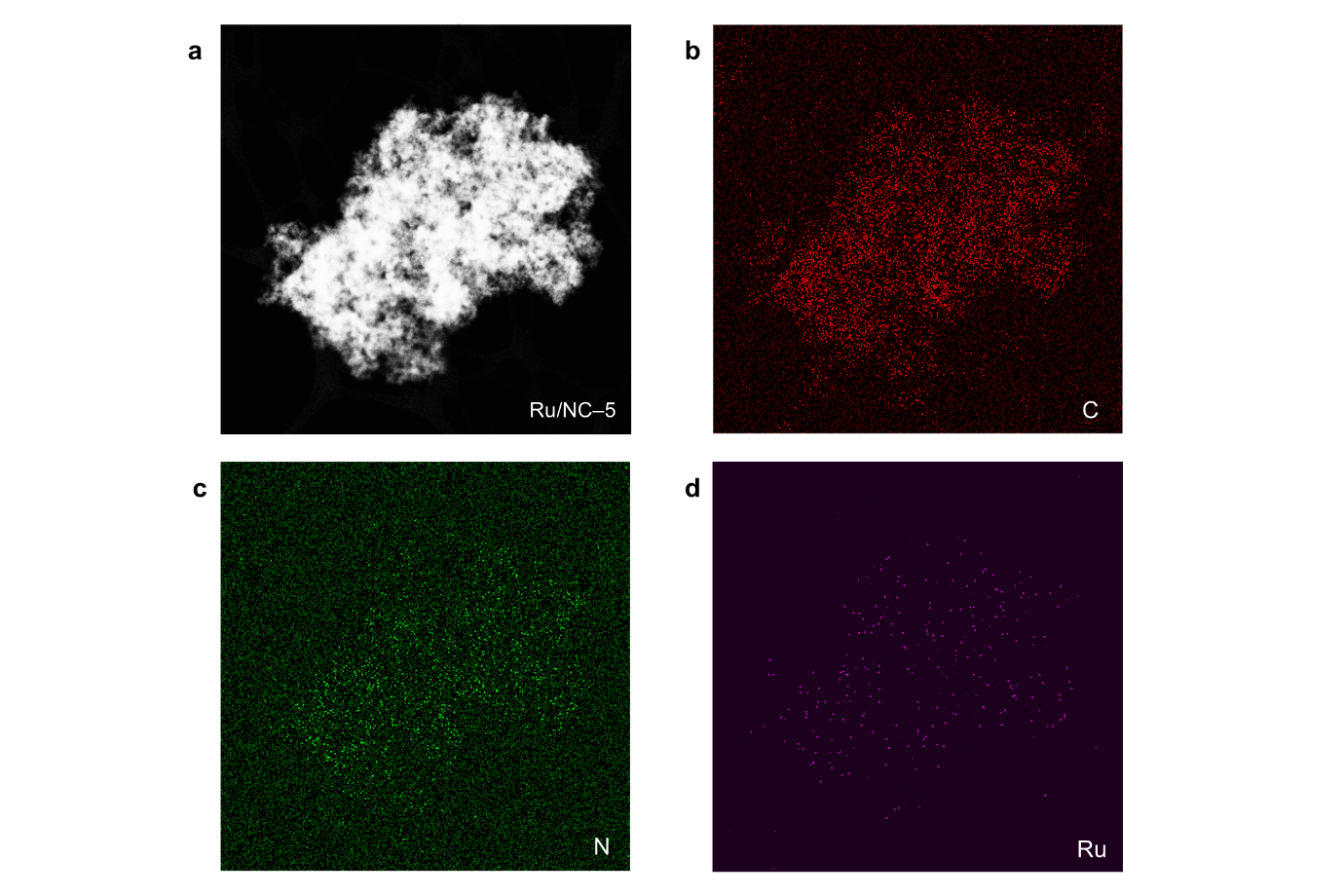


**Figure S6.** a) HAADF-STEM image of the Ru/NC–5. Corresponding EDS elementary mapping images. b) C; c) N; d) Ru.


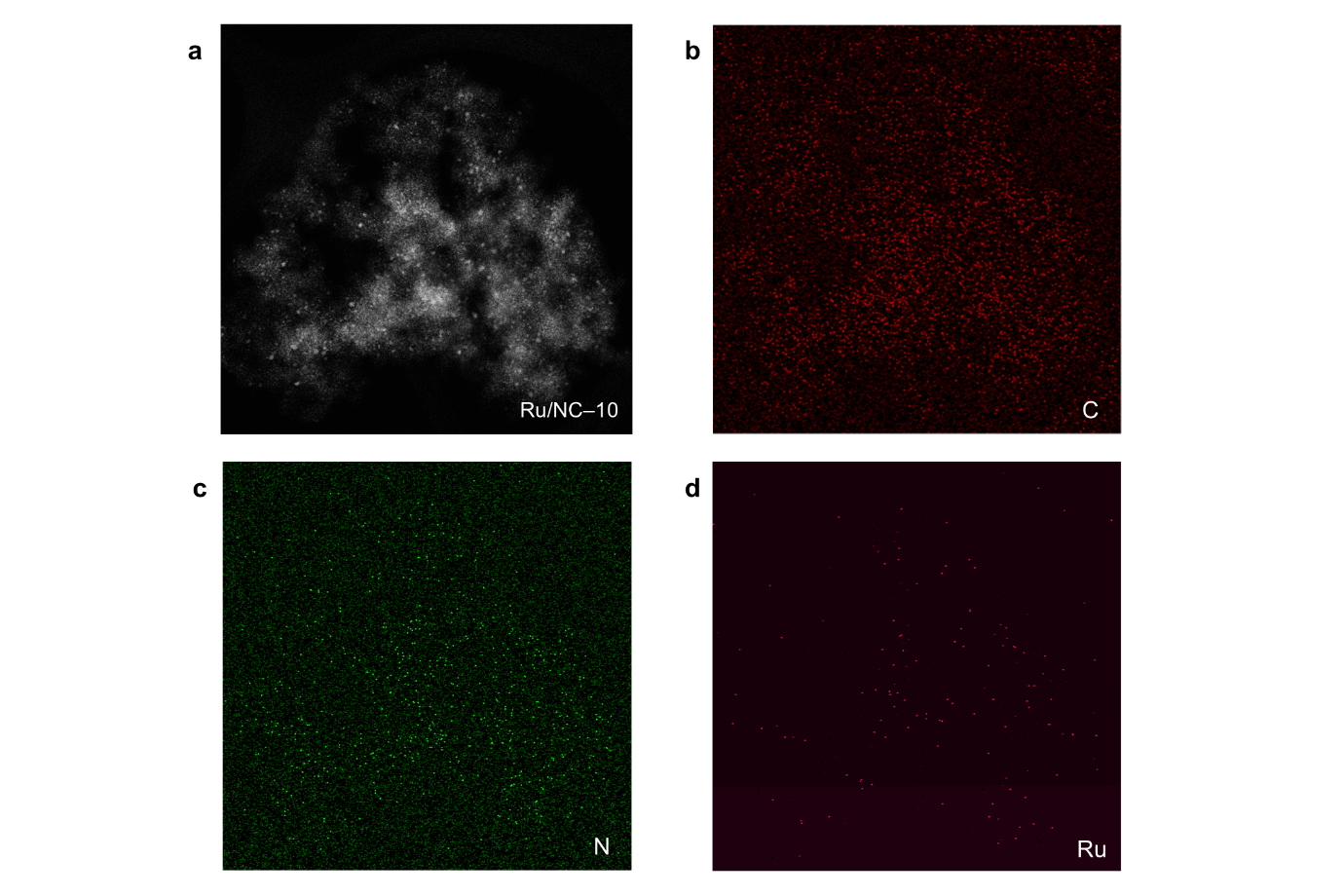


**Figure S7.** a) HAADF-STEM image of the Ru/NC–10. Corresponding EDS elementary mapping images. b) C; c) N; d) Ru.


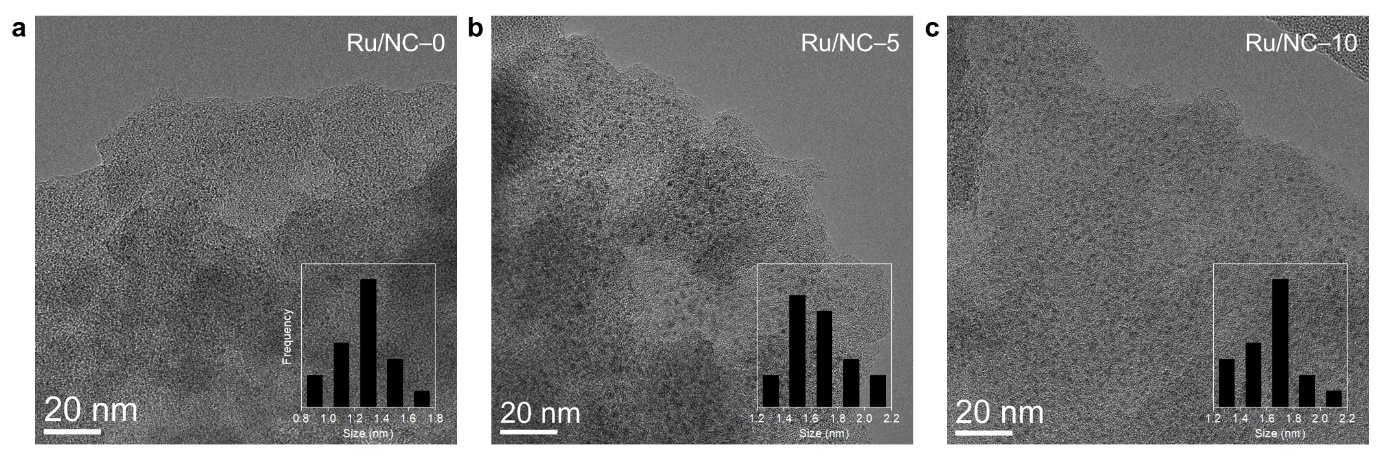


**Figure S8.** HR-TEM images. a) Ru/NC–0; b) Ru/NC–5; c) Ru/NC–10. Inset in each image is a corresponding cluster size distribution of the Ru_NC_.

**
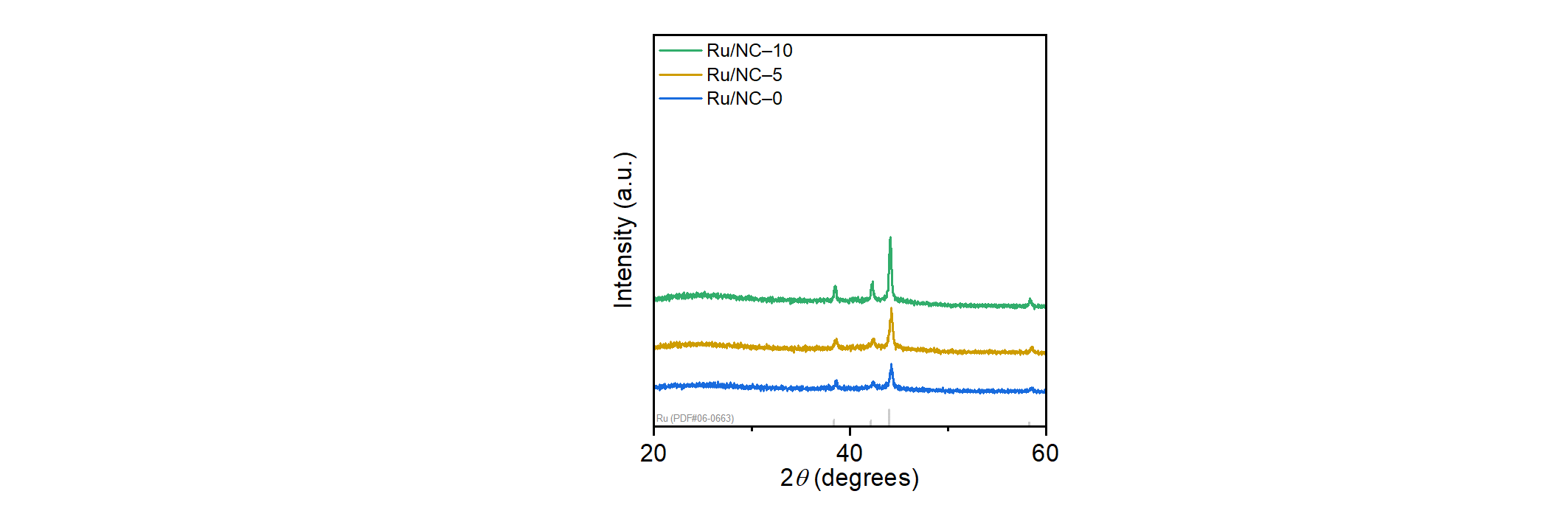
**

**Figure S9.** HP-XRD patterns of Ru/NC–0, Ru/NC–5, and Ru/NC–10.

**
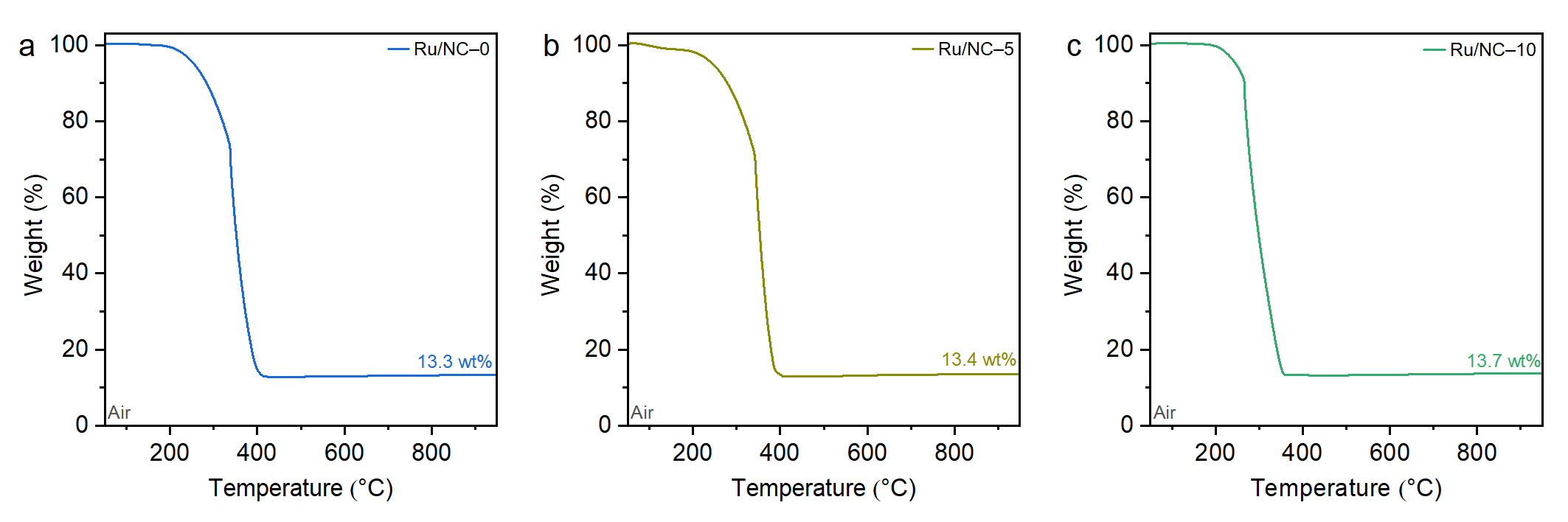
**

**Figure S10.** TGA thermograms obtained at a ramping rate of 10 °C min^−1^ under air atmosphere. a) Ru/NC–0; b) Ru/NC–5; c) Ru/NC–10.


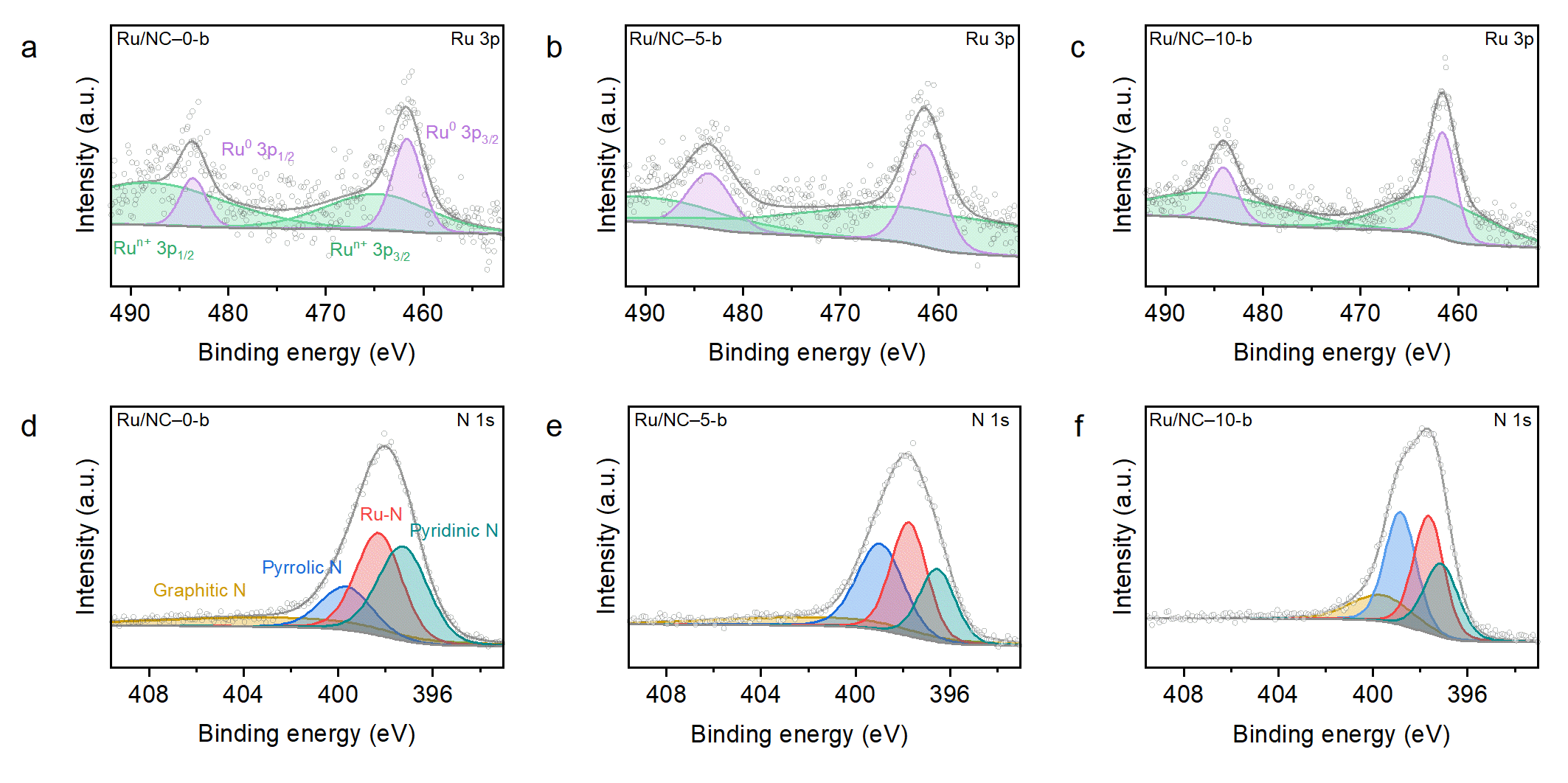


**Figure S11.** High-resolution XPS survey spectra of Ru 3p.^[1]^ a) Ru/NC–0-b; b) Ru/NC–5-b; c) Ru/NC–10-b. High-resolution XPS survey spectra of N 1s.^[1]^ d) Ru/NC–0-b; e) Ru/NC–5-b; f) Ru/NC–10-b.

**
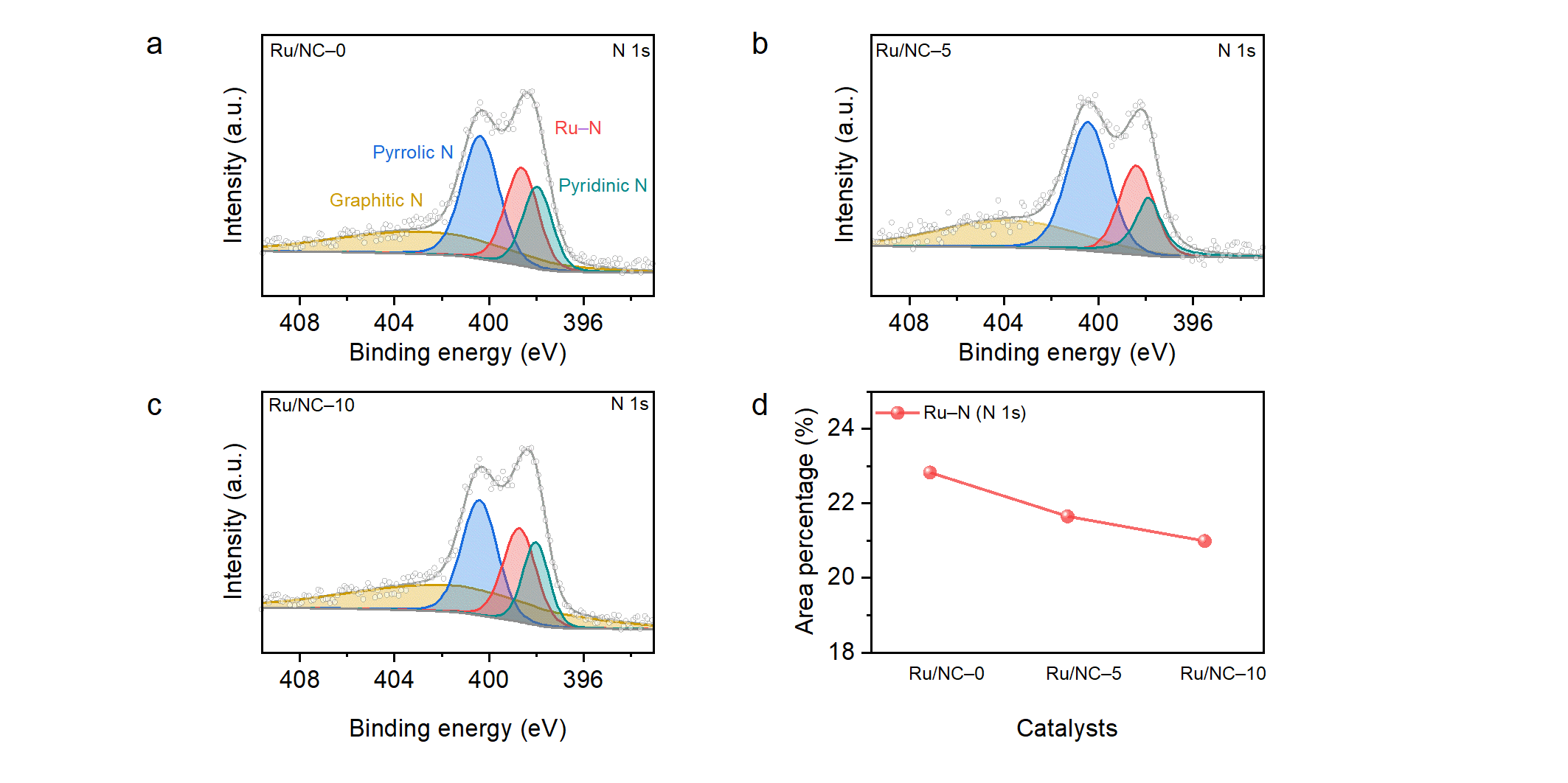
**

**Figure S12.** High-resolution XPS survey spectra of N 1s.^[1]^ a) Ru/NC–0; b) Ru/NC–5; c) Ru/NC–10. d) XPS peak area ratio of Ru-N (N 1s) across the Ru/NC–0, Ru/NC–5, and Ru/NC–10.^[1]^


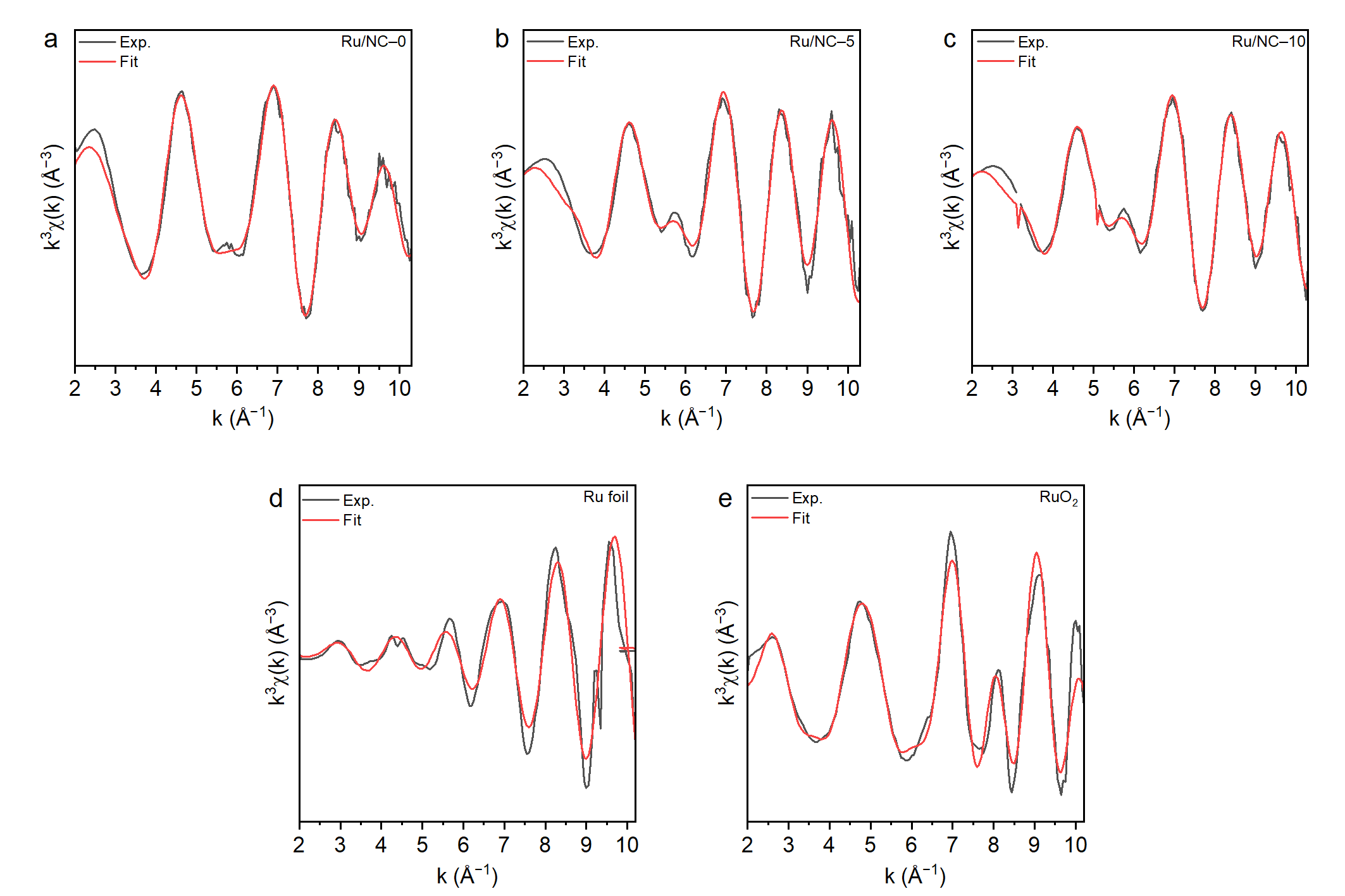


**Figure S13.** The k spaces fitting curves. a) Ru/NC–0; b) Ru/NC–5; c) Ru/NC–10; d) Ru foil; e) RuO_2_.


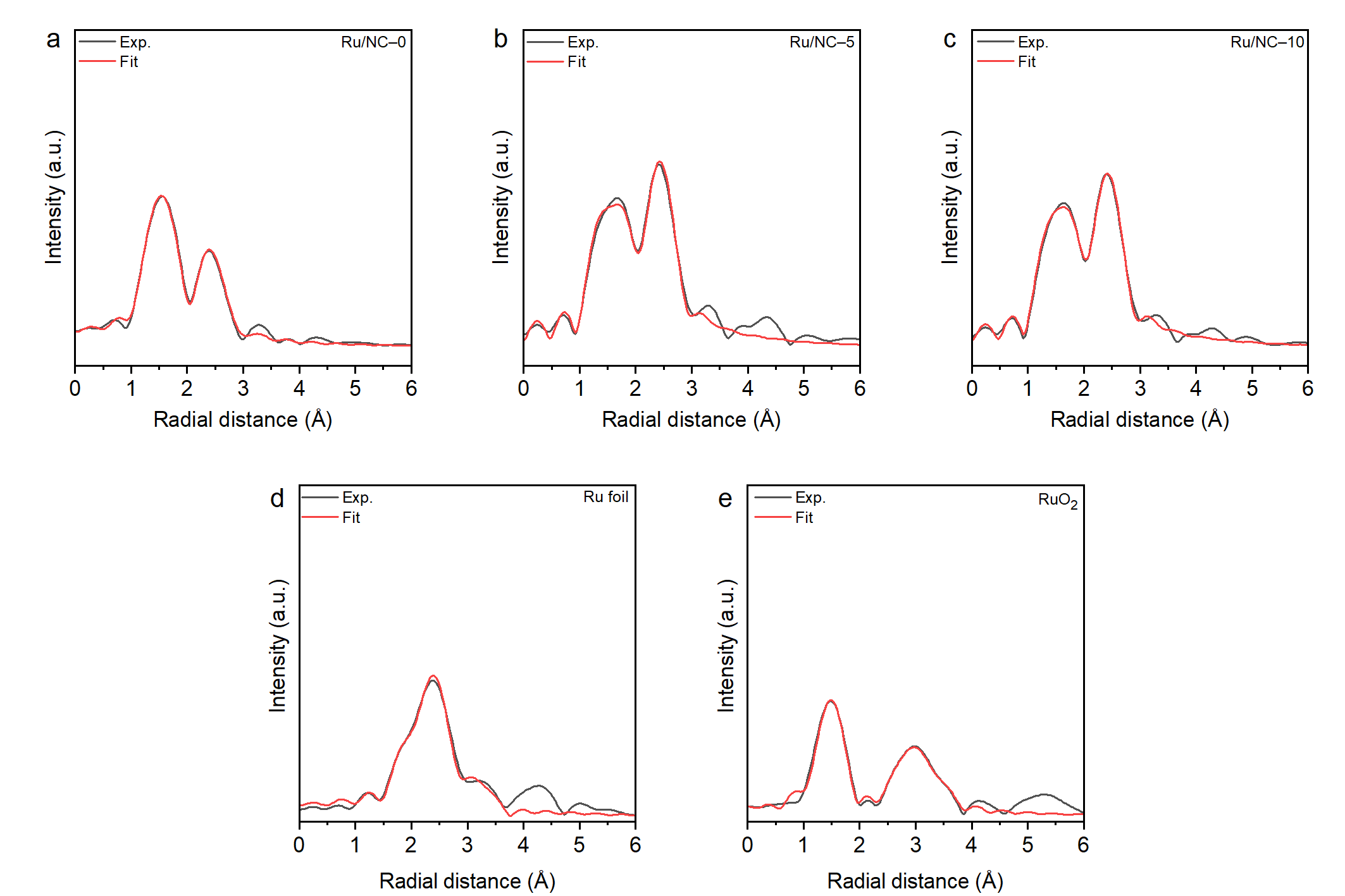


**Figure S14.** The r spaces fitting curves. a) Ru/NC–0; b) Ru/NC–5; c) Ru/NC–10; d) Ru foil; e) RuO_2_.


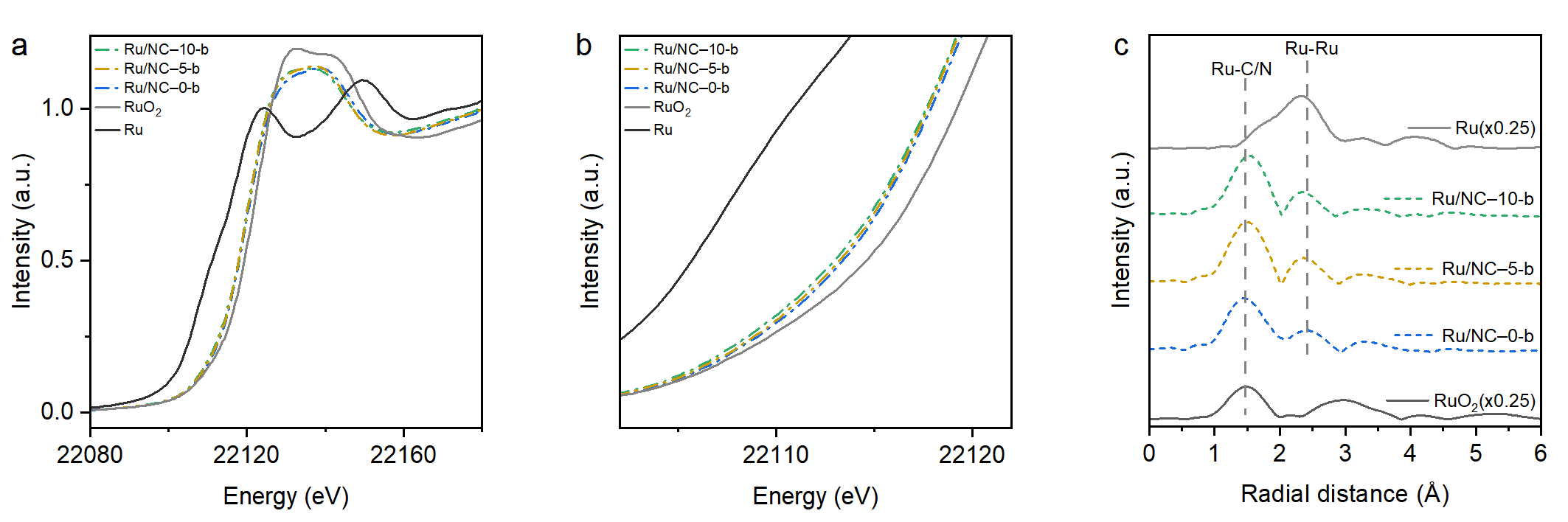


**Figure S15.** a) Ru K-edge XANES spectra. b) Magnified Ru K-edge XANES spectra. c) Fourier-transformed Ru K-edge EXAFS spectra for Ru/NC–0-b, Ru/NC–5-b, Ru/NC–10-b, Ru foil and RuO_2_.


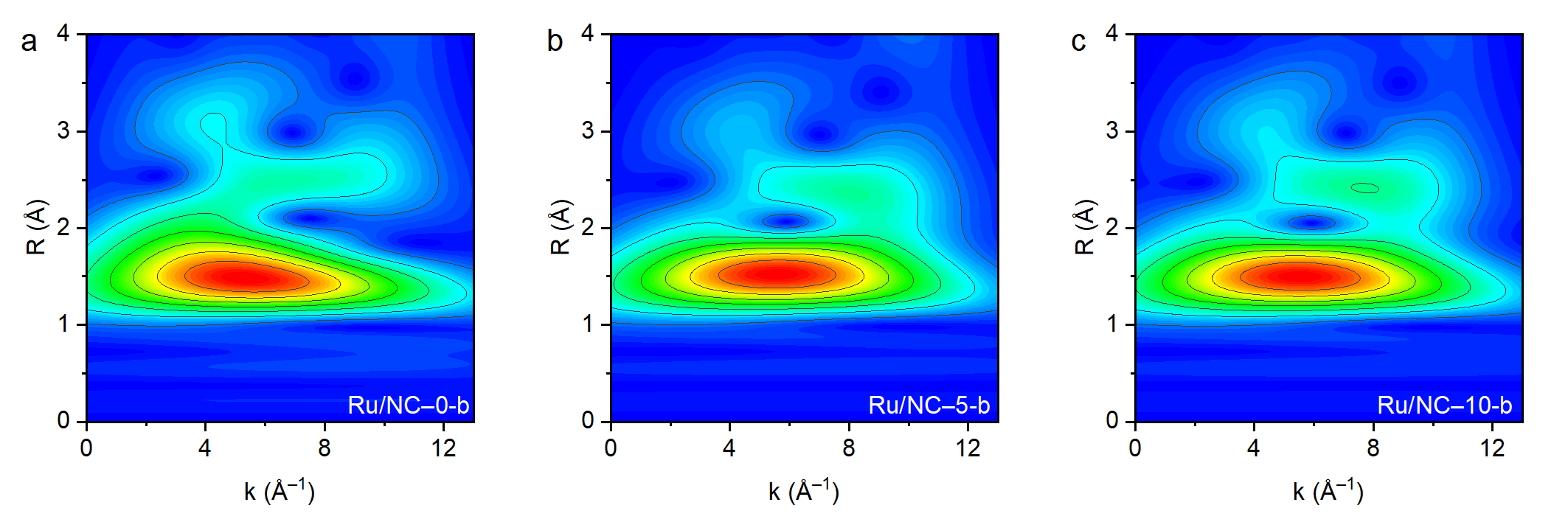


**Figure S16.** Wavelet transforms of the k^3^-weighted EXAFS signs. a) Ru/NC–0-b; b) Ru/NC–5-b; c) Ru/NC–10-b.


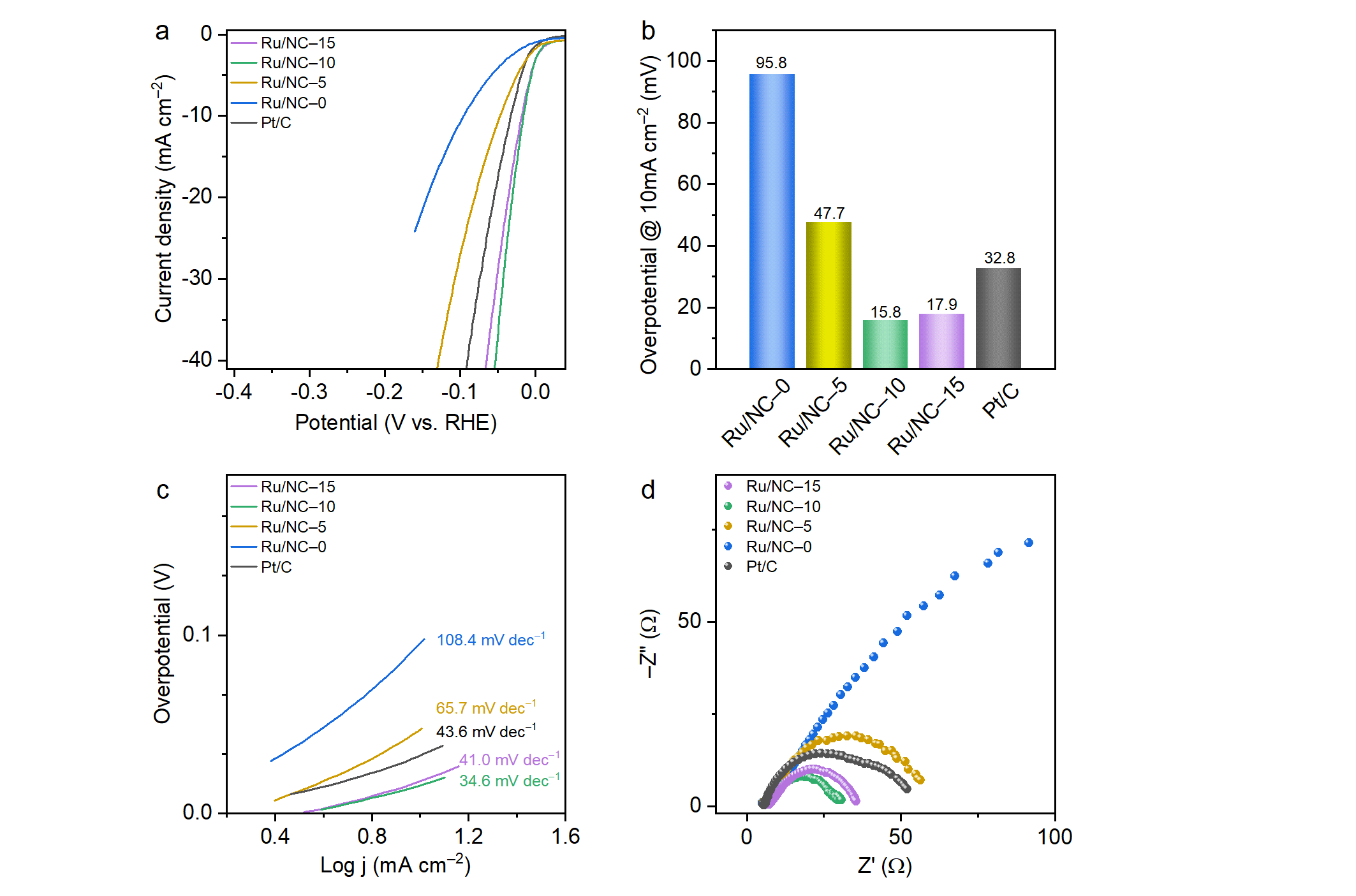


**Figure S17.** Electrochemical analyses of Ru/NC–0, Ru/NC–5, Ru/NC–10, Ru/NC–15 and Pt/C in 1.0 ᴍ aq. KOH electrolyte. a) Polarization curves. b) Overpotential values at 10 mA cm^–2^. c) Tafel slopes. d) Charge resistance values.


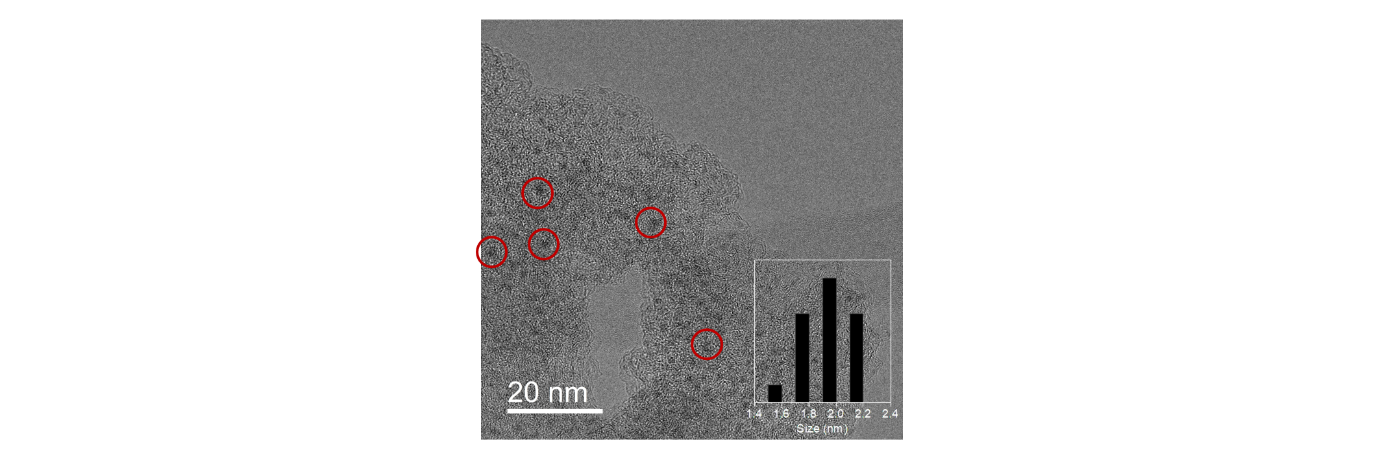
**Figure S18.** HR-TEM image of Ru/NC–15. Inset in each image is a corresponding cluster size distribution of the Ru_NC_. Red circles indicate larger Ru nanoparticles compared to those in the baseline sample Ru/NC–10.

**
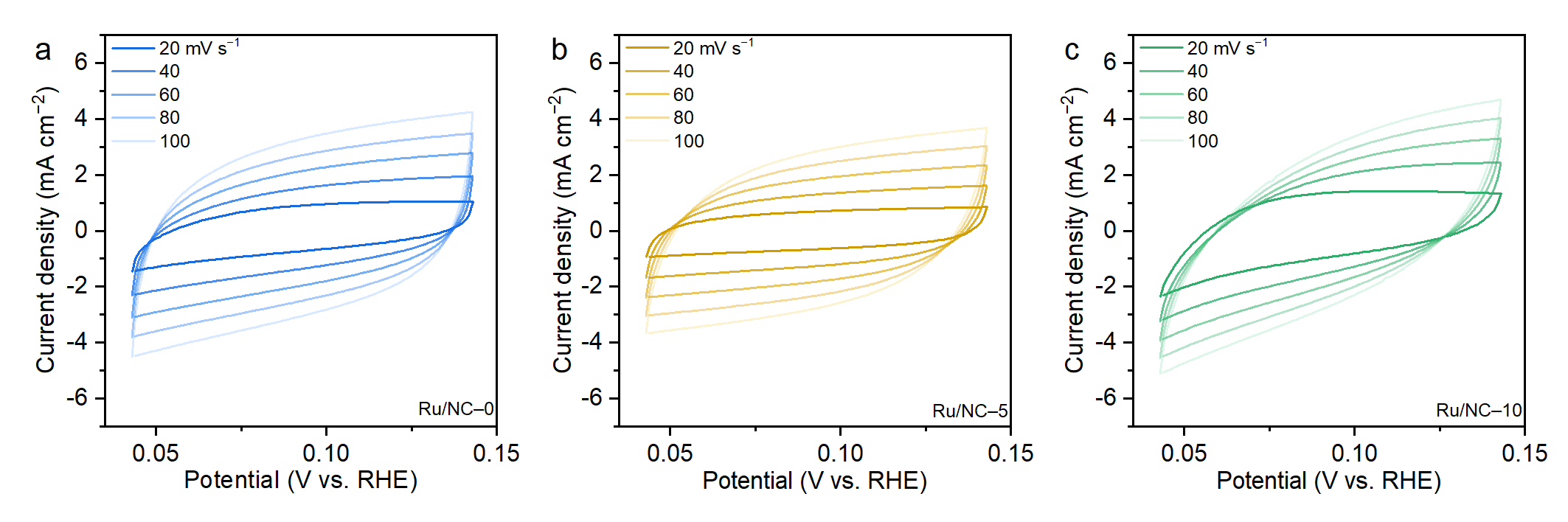
**

**Figure S19.** Double-layer capacitance curves in 1.0 ᴍ aq. KOH solution. a) Ru/NC–0; b) Ru/NC–5; c) Ru/NC–10.

**
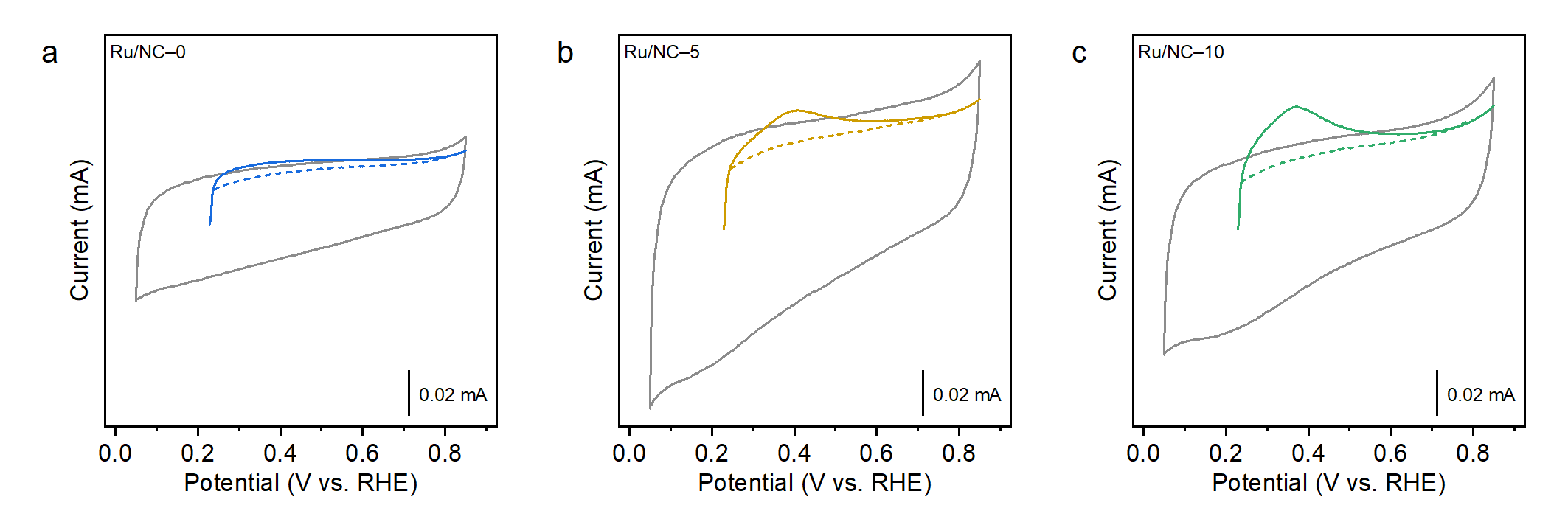
**

**Figure S20.** Cu underpotential deposition curves in 0.5 ᴍ aq. H_2_SO_4_ in the absence and presence of 5 mM CuSO_4_. a) Ru/NC–0; b) Ru/NC–5; c) Ru/NC–10.

**
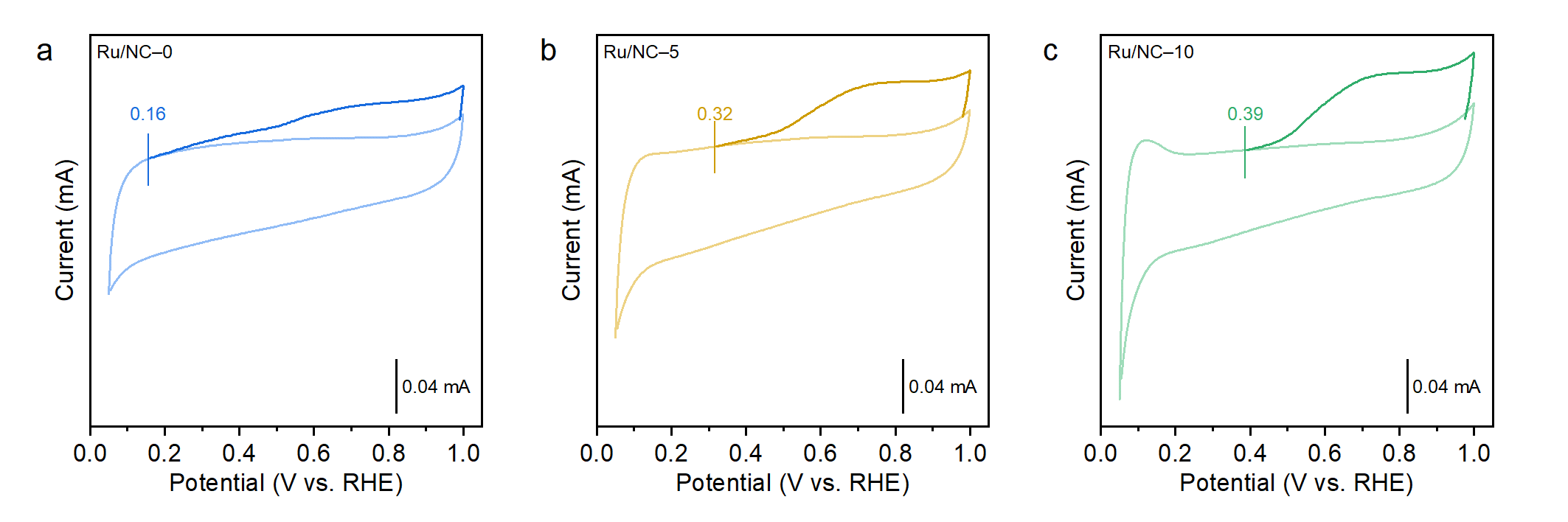
**

**Figure S21.** CO stripping curves in 1.0 ᴍ aq. KOH in the absence and presence of CO/Ar mixture gas. a) Ru/NC–0; b) Ru/NC–5; c) Ru/NC–10.

**
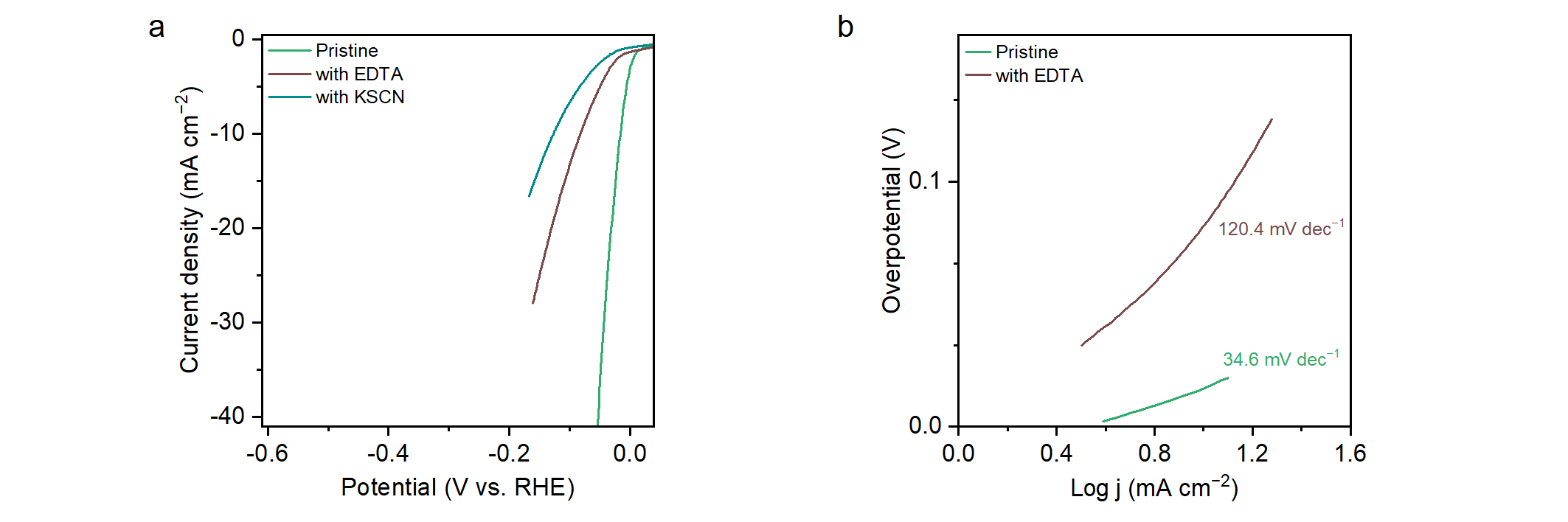
**

**Figure S22.** a) LSV curves of Ru/NC–10 in 1.0 ᴍ aq. KOH solution: pristine (without additives), with EDTA, and with KSCN. b) Tafel slopes before and after adding EDTA into the 1.0 ᴍ aq. KOH solution.


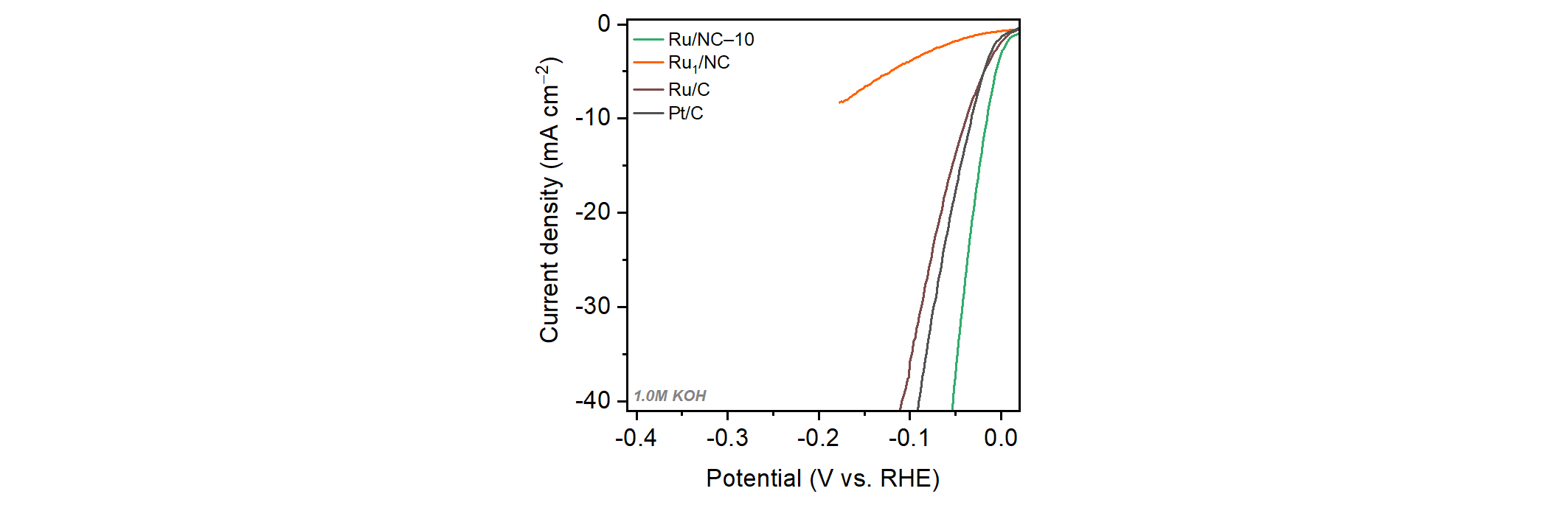


**Figure S23.** LSV curves of Ru/NC–10, Ru_1_/NC, Ru/C, and Pt/C in the 1.0 ᴍ aq. KOH solution.


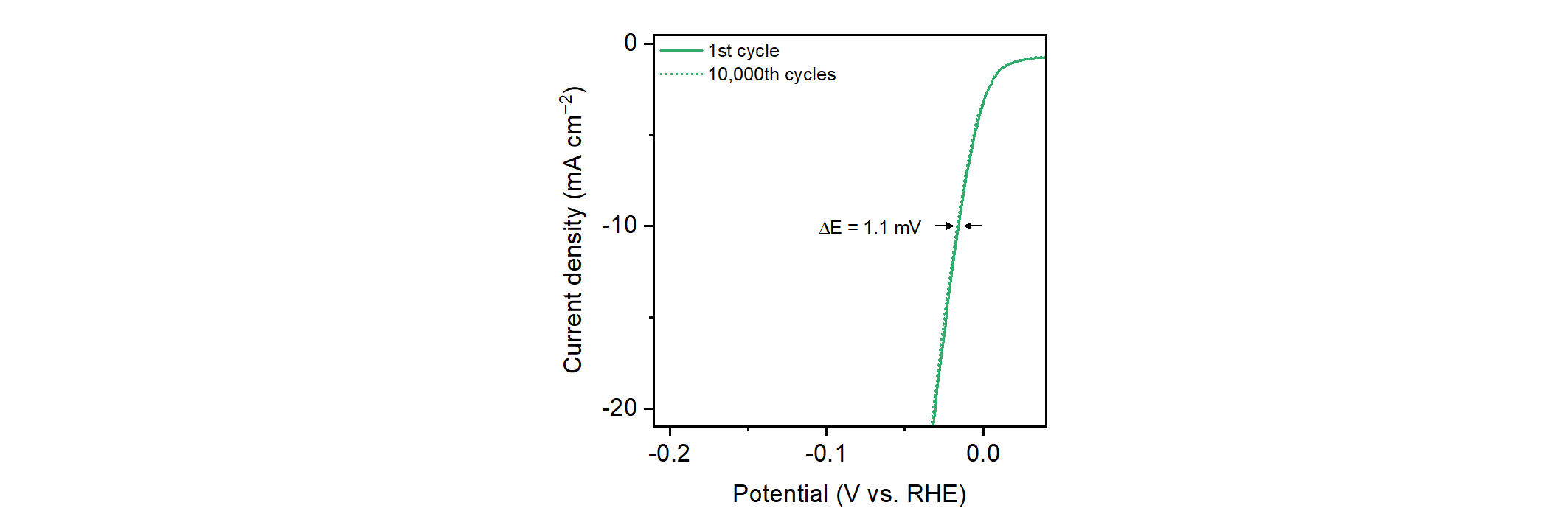


**Figure S24.** Stability test measured after 10,000 CV cycles of Ru/NC–10 in 1.0 ᴍ aq. KOH solution.


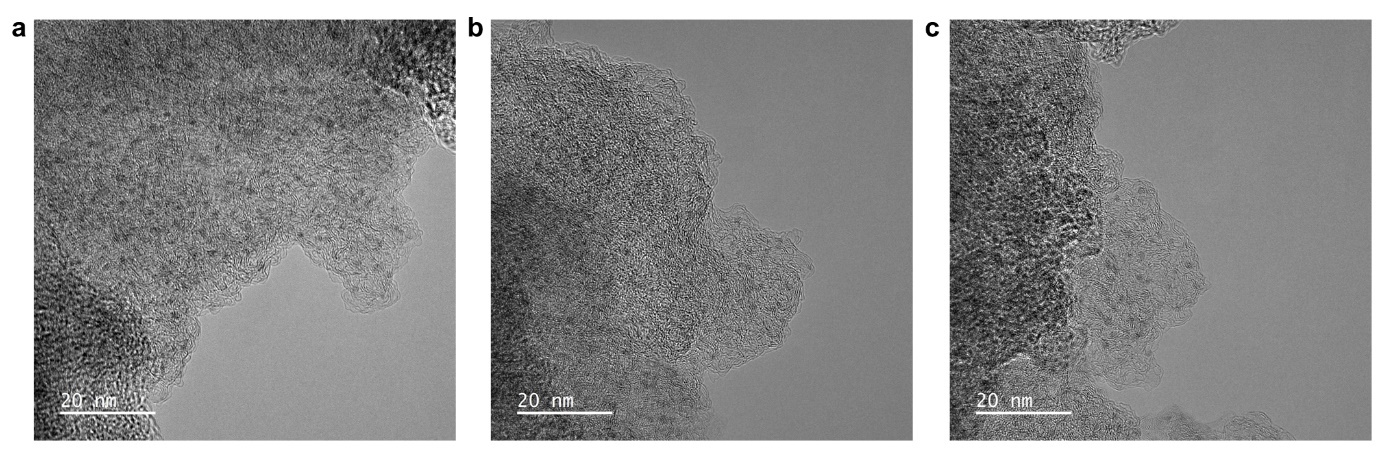
 **Figure S25.** a-c) HR-TEM images obtained at different parts of Ru/NC–10 after the stability test.





**Figure S26.** HP-XRD patterns of Ru/NC–10 after stability test.





**Figure S27.** High-resolution XPS survey spectra of N 1s for Ru/NC–10 after stability test.^[1]^


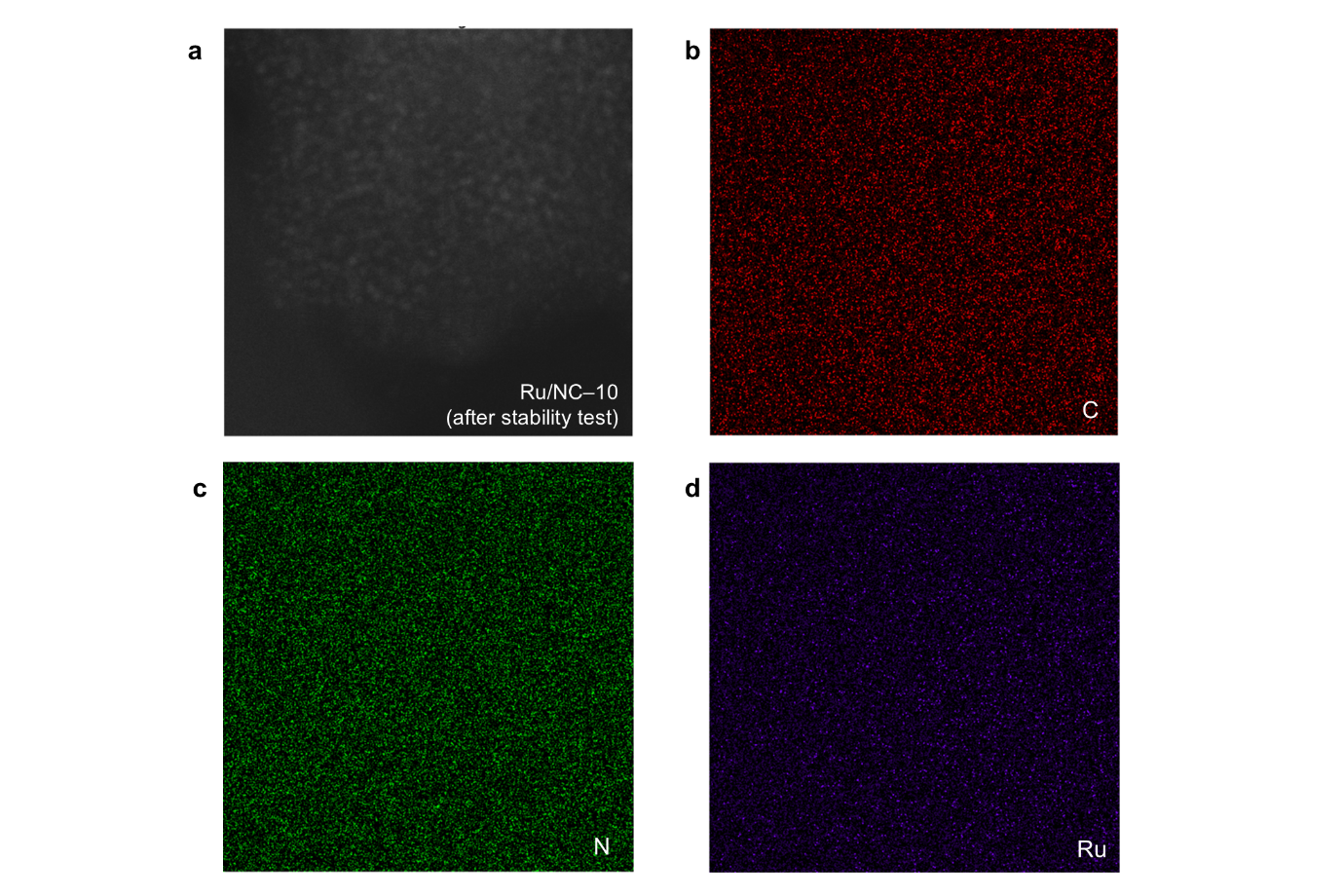


**Figure** **S28.** a) HAADF-STEM image of the Ru/NC–10 after stability test. Corresponding EDS elementary mapping images. b) C; c) N; d) Ru.


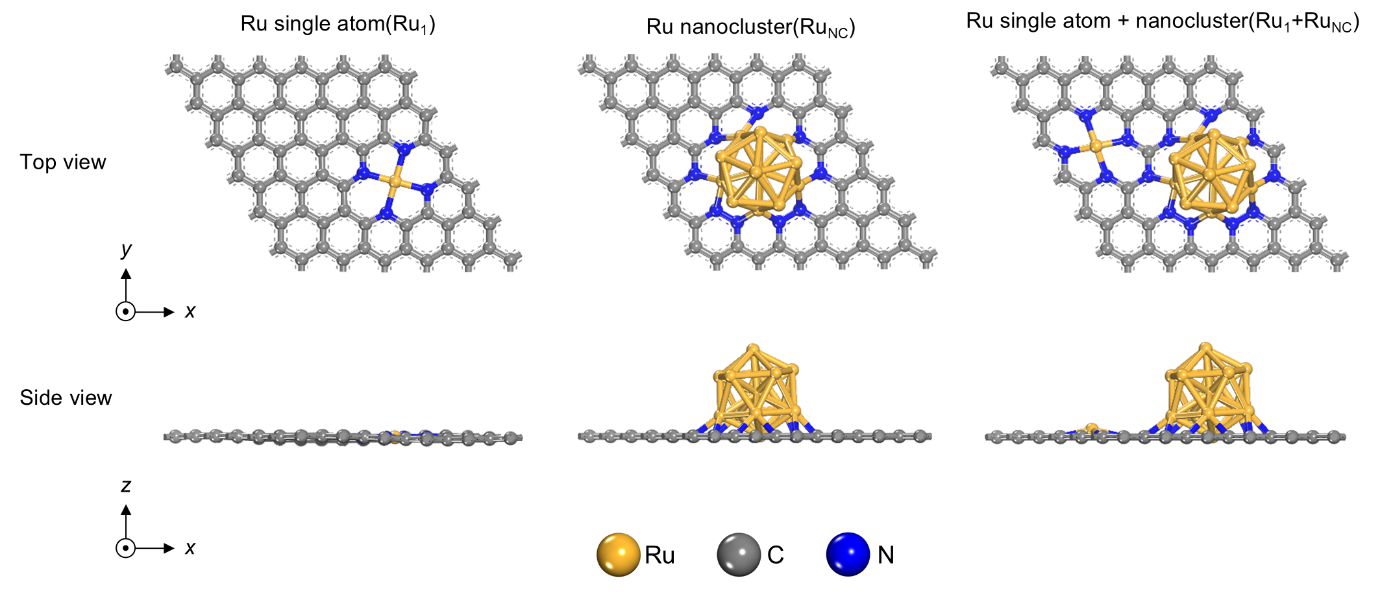


**Figure S29.** Surface models with a Ru_1_ site, Ru_NC_ site, and coexisting Ru active site (Ru_1_ + Ru_NC_). Note that the Ru­_1_ site, Ru_NC_ site, and coexisting Ru active site are denoted as Ru_1_, Ru_NC,_ and Ru_1_+Ru_NC,_ respectively.


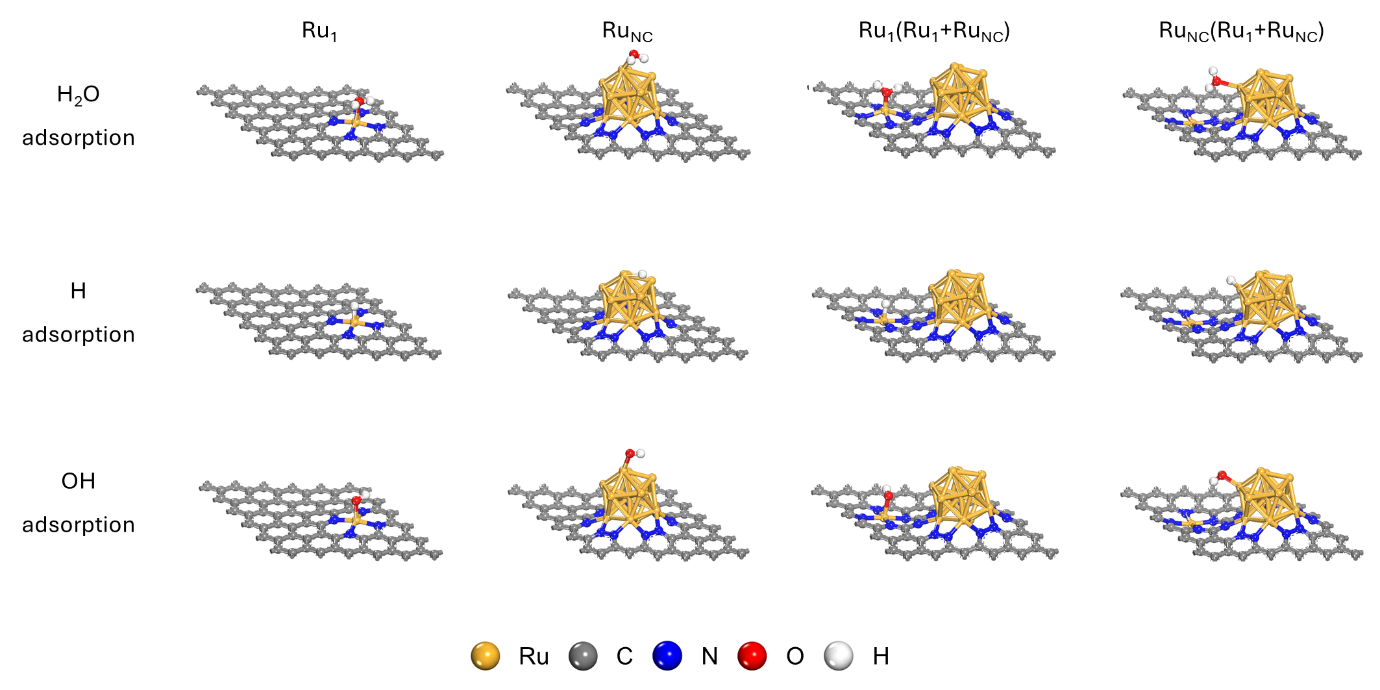


**Figure S30.** Optimized structures of H*, H_2_O, and OH* adsorption for Ru_1_, Ru_NC,_ Ru_1_(Ru_1_+Ru_NC_), and Ru_NC_(Ru_1_+Ru_NC_).


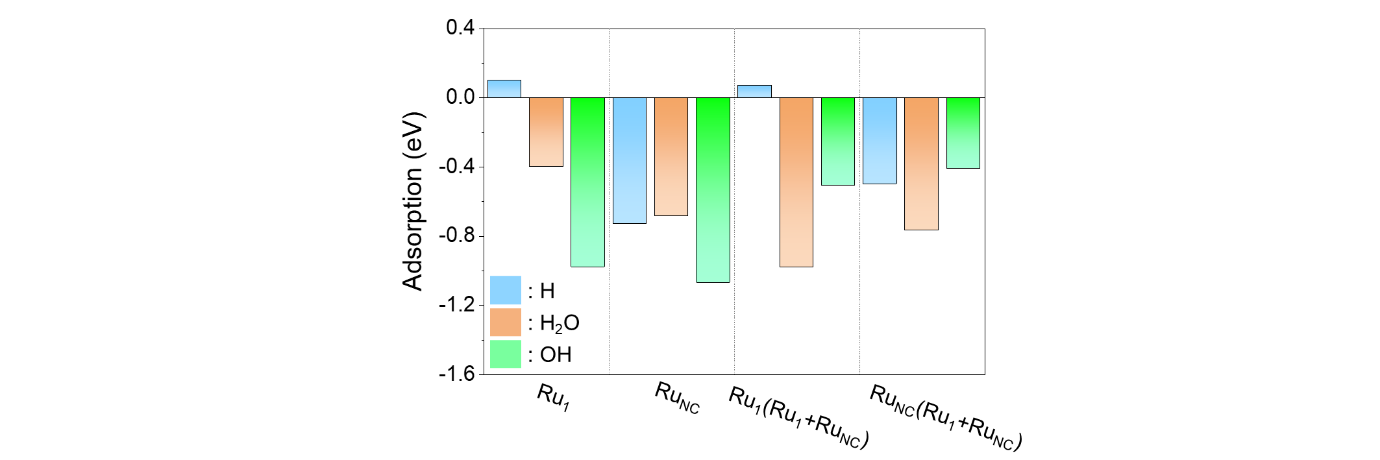


**Figure S31.** H*, H_2_O, and OH* adsorption energies for Ru_1_, Ru_NC,_ Ru_1_(Ru_1_+Ru_NC_), and Ru_NC_(Ru_1_+Ru_NC_).


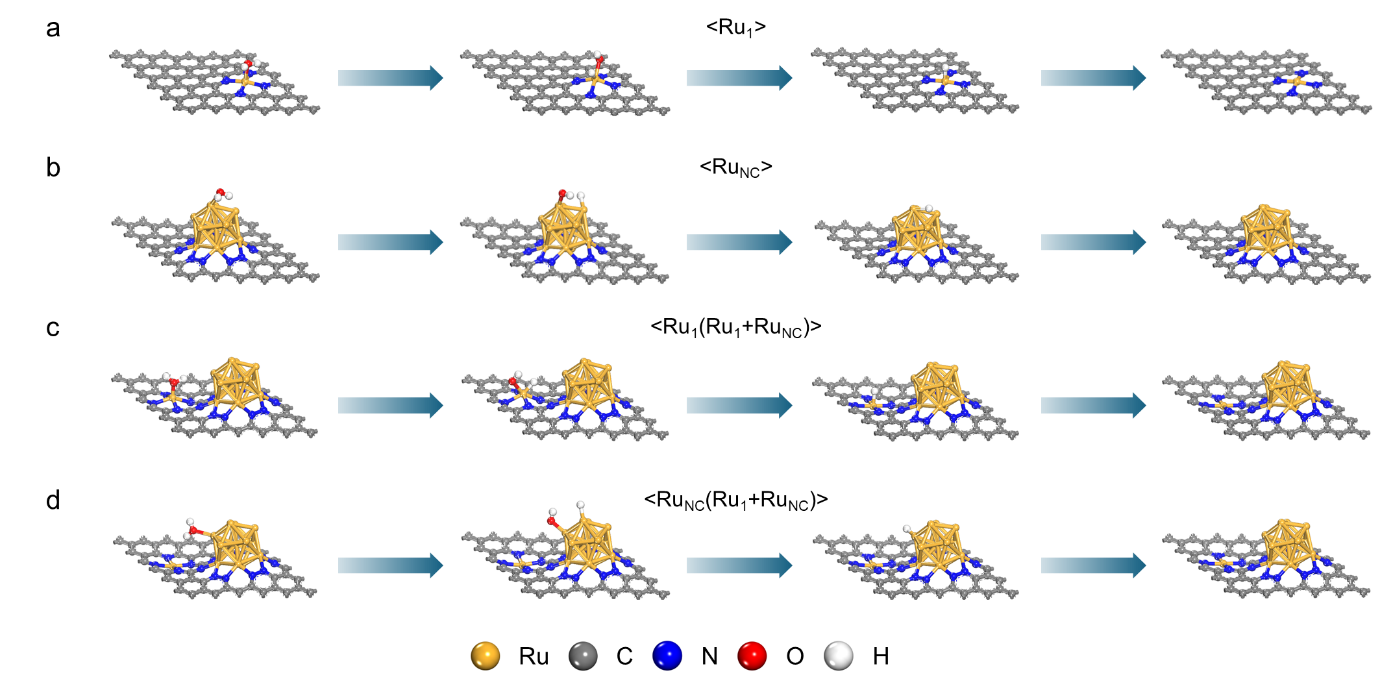


**Figure S32.** Schematic diagrams of the alkaline HER mechanism: a) only Ru_1_ site; b) only Ru_NC_ site; c) Ru_1_ site in a coexisting system (Ru_1_(Ru_1_+Ru_NC_)); d) Ru_NC_ site in a coexisting system (Ru_NC_(Ru_1_+Ru_NC_)).


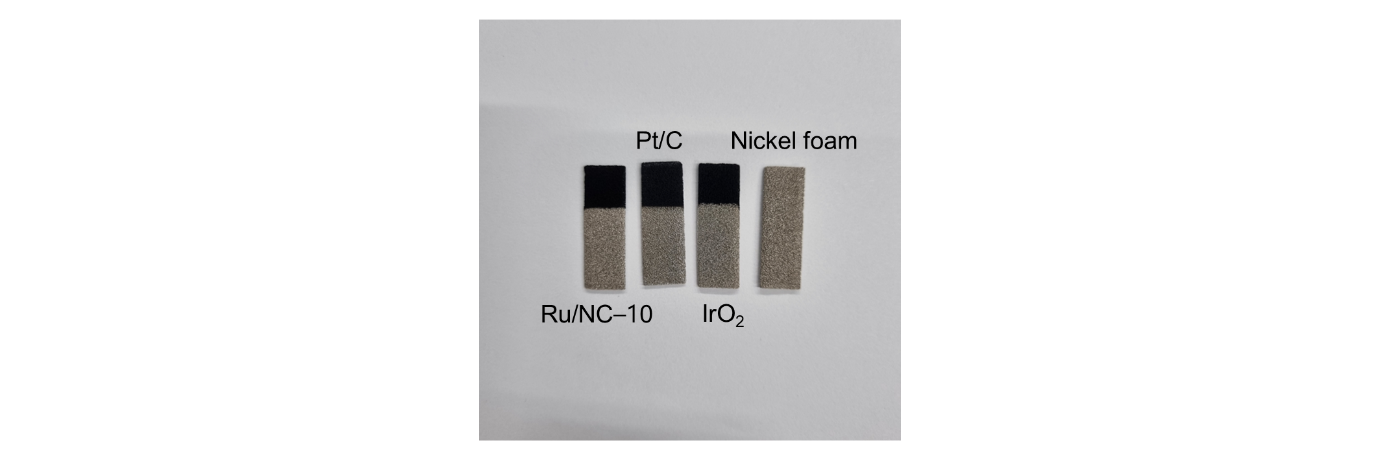
 **Figure S33.** Digital photograph of uniformly coated electrodes.


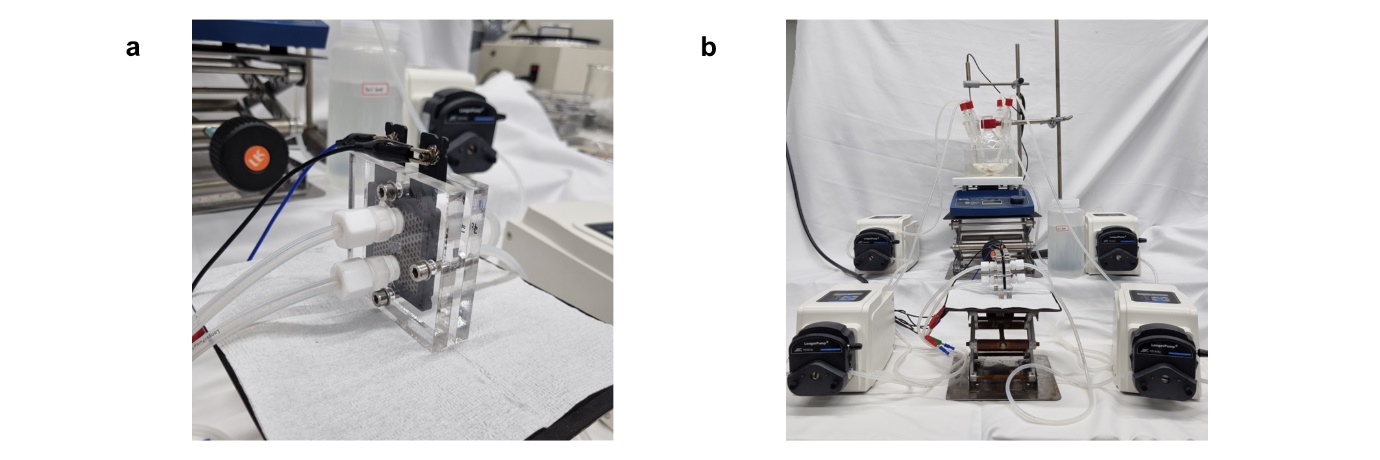


**Figure S34.** Digital photographs of alkaline AEMWE setup. a) AEMWE single cell; b) Full AEMWE setup.

**Tables**

**Table S1.** Results of elemental analyses revealing Ru/NC–X composition

| **Samples** | **O (wt%)** | **N (wt%)** | **C (wt%)** | **H (wt%)** | **Ru (wt%)^a^** | **Total** |
| --- | --- | --- | --- | --- | --- | --- |
| **Ru/NC–0** | 3.42 | 7.10 | 72.99 | 1.87 | 14.62^a^ | 100 |
| **Ru/NC–5** | 3.78 | 7.05 | 73.97 | 1.92 | 13.28^a^ | 100 |
| **Ru/NC–10** | 3.73 | 6.36 | 74.76 | 1.74 | 13.41^a^ | 100 |

^a^ The calculated difference between the sum of the carbon (C), oxygen (O), and hydrogen (H) contents starting at 100 wt% closely matches the data obtained from thermogravimetric analysis (TGA) in air atmosphere.

**Table S2.** Summary of XPS Ru 3p data from fitting calculations for Ru/NC–X-b and Ru/NC–X

| **Samples** | **Assignment** | **Peak (eV)** | **Peak area (%)** |
| --- | --- | --- | --- |
| **Ru/NC–0-b** | Ru 3p_2/3_ (Ru^0^) | 461.6 | 28.07 |
|  | Ru 3p_2/3_ (Ru^n+^) | 464.6 | 71.93 |
| **Ru/NC–5-b** | Ru 3p_2/3_ (Ru^0^) | 461.5 | 30.14 |
|  | Ru 3p_2/3_ (Ru^n+^) | 464.5 | 69.86 |
| **Ru/NC–10-b** | Ru 3p_2/3_ (Ru^0^) | 461.5 | 34.65 |
|  | Ru 3p_2/3_ (Ru^n+^) | 464.1 | 65.35 |
| **Ru/NC–0** | Ru 3p_2/3_ (Ru^0^) | 462.0 | 46.04 |
|  | Ru 3p_2/3_ (Ru^n+^) | 464.2 | 53.96 |
| **Ru/NC–5** | Ru 3p_2/3_ (Ru^0^) | 462.0 | 50.55 |
|  | Ru 3p_2/3_ (Ru^n+^) | 464.4 | 49.45 |
| **Ru/NC–10** | Ru 3p_2/3_ (Ru^0^) | 462.1 | 55.26 |
|  | Ru 3p_2/3_ (Ru^n+^) | 464.3 | 44.74 |

**Table S3.** EXAFS fitting parameters^a^ for the Ru K-edge of Ru/NC–X

| **Sample** | **Path** | **N^b^** | **R (Å)^c^** | **σ^2^×10^3^ (Å^2^)^d^** | **ΔE_0_ (eV)^e^** | **R factor** |
| --- | --- | --- | --- | --- | --- | --- |
| **Ru foil** | Ru-Ru | 12 | 2.65±0.03 | 2.1±0.2 | -5.7±1.3 | 0.006 |
| **RuO_2_** | Ru-O | 6 | 1.94±0.03 | 2.5±0.5 | 3.3±1.6 | 0.005 |
| **Ru/NC–0** | Ru-N | 4.8±0.4 | 2.03±0.01 | 6.7±0.9 | 2.5±0.8 | 0.001 |
|  | Ru-Ru | 1.7±0.3 | 2.69±0.01 | 6.1±1.3 |  |  |
| **Ru/NC–5** | Ru-N | 4.2±0.7 | 2.02±0.01 | 5.3±1.9 | 1.5±1.4 | 0.003 |
|  | Ru-Ru | 2.7±0.6 | 2.68±0.05 | 4.2±1.5 |  |  |
| **Ru/NC–10** | Ru-N | 3.8±0.4 | 2.01±0.01 | 4.2±1.2 | 1.1±0.9 | 0.001 |
|  | Ru-Ru | 2.8±0.4 | 2.68±0.06 | 4.9±1.0 |  |  |

^a^ S_o_^2^ was fixed at 0.8.^[2]^ ^b^ *N*: coordination numbers; ^c^ *R*: bond distance; ^d^ *σ^2^*: Debye-Waller factors; ^e^ *ΔE_0_*: the inner potential correction. R factor: goodness of fit. according to the experimental EXAFS fit.

**Table S4.** Performance comparison of Ru/NC–10 with other reported Ru-based alkaline HER catalysts

| **Catalyst** | **Overpotential**  **@ 10 mA cm^-2^ (mV)** | **Electrolyte**  **condition** | **Reference** |
| --- | --- | --- | --- |
| **Ru/NC–10** | **15.8** | **1.0 ᴍ KOH** | **This work** |
| R-NiRu | 16.0 | 1.0 ᴍ KOH | ^[3]^ |
| Ru_1,n_-NC | 14.8 | 1.0 ᴍ KOH | ^[4]^ |
| Ru/NC | 25.0 | 1.0 ᴍ KOH | ^[5]^ |
| Ru/MoO_2_ | 16.0 | 1.0 ᴍ KOH | ^[6]^ |
| Ru/RuO_2_-180 | 38.0 | 1.0 ᴍ KOH | ^[7]^ |
| NiCoRu_0.2_/SP | 59.0 | 1.0 ᴍ KOH | ^[8]^ |
| Ru_n_-Ru_s_/NC | 37.0 | 1.0 ᴍ KOH | ^[9]^ |
| CC@WS_2_/Ru-450 | 32.1 | 1.0 ᴍ KOH | ^[10]^ |
| Ni_5_P_4_-Ru | 54.0 | 1.0 ᴍ KOH | ^[11]^ |
| Pt–Ru/RuO_2_ | 18.0 | 1.0 ᴍ KOH | ^[12]^ |
| Ru-Fe_3_O_4_/C | 11.0 | 1.0 ᴍ KOH | ^[13]^ |
| RuO_2_-300Ar | 17.0 | 1.0 ᴍ KOH | ^[14]^ |
| Ru@MoO(S)_3_ | 30.0 | 1.0 ᴍ KOH | ^[15]^ |
| Ir-Ru@C | 13.0 | 1.0 ᴍ KOH | ^[16]^ |
| P–Ru/C | 31.0 | 1.0 ᴍ KOH | ^[17]^ |
| Ru@WNO-C | 24.0 | 1.0 ᴍ KOH | ^[18]^ |
| Ru–Ni(OH)_2_/NF | 15.0 | 1.0 ᴍ KOH | ^[19]^ |
| Ru-1.0 | 13.0 | 1.0 ᴍ KOH | ^[20]^ |
| Cu@Cu_3_P-Ru/CCG-500 | 33.0 | 1.0 ᴍ KOH | ^[21]^ |
| Ru/np-MoS_2_ | 30.0 | 1.0 ᴍ KOH | ^[22]^ |
| Co_5_Ru_1_@NCNT/PF | 28.0 | 1.0 ᴍ KOH | ^[23]^ |
| RuP@RuP_2_/C | 11.6 | 1.0 ᴍ KOH | ^[24]^ |
| Ru/Zn-N-C | 17.6 | 1.0 ᴍ KOH | ^[25]^ |
| c-RP/IP HNT/C | 23.2 | 1.0 ᴍ KOH | ^[26]^ |
| Ru-Cu-2 | 33.0 | 1.0 ᴍ KOH | ^[27]^ |
| Ru/CoSA/CNT | 15.0 | 1.0 ᴍ KOH | ^[28]^ |
| Mo–Ru NSAs | 16.0 | 1.0 ᴍ KOH | ^[29]^ |
| Ru-SAs@MoC/NCFs | 16.0 | 1.0 ᴍ KOH | ^[30]^ |
| 1.38 nm Ru NPs | 27.0 | 1.0 ᴍ KOH | ^[31]^ |
| c/a-Ru/VO_x_-500 | 33.0 | 1.0 ᴍ KOH | ^[32]^ |
| Ru@NCN | 36.0 | 1.0 ᴍ KOH | ^[33]^ |

**References**

[1] a)C. Mu, H. Xin, Q. Luo, Y. Li, F. Ma, *Journal of Materials Chemistry A* **2023**, 11, 7016; b)M. Tamtaji, M. G. Kim, J. WANG, P. R. Galligan, H. Zhu, F.-F. Hung, Z. Xu, Y. Zhu, Z. Luo, W. A. Goddard, G. Chen, *Advanced Science* **2024**, 11, 2309883.

[2] T. Vitova, S. Mangold, C. Paulmann, M. Gospodinov, V. Marinova, B. Mihailova, *Physical Review B* **2014**, 89, 144112.

[3] X. Chen, J. Wan, J. Wang, Q. Zhang, L. Gu, L. Zheng, N. Wang, R. Yu, *Advanced Materials* **2021**, 33, 2104764.

[4] X. Chen, X.-T. Wang, J.-B. Le, S.-M. Li, X. Wang, Y.-J. Zhang, P. Radjenovic, Y. Zhao, Y.-H. Wang, X.-M. Lin, J.-C. Dong, J.-F. Li, *Nature Communications* **2023**, 14, 5289.

[5] Y. Zhu, K. Fan, C.-S. Hsu, G. Chen, C. Chen, T. Liu, Z. Lin, S. She, L. Li, H. Zhou, Y. Zhu, H. M. Chen, H. Huang, *Advanced Materials* **2023**, 35, 2301133.

[6] H. Li, K. Liu, J. Fu, K. Chen, K. Yang, Y. Lin, B. Yang, Q. Wang, H. Pan, Z. Cai, H. Li, M. Cao, J. Hu, Y.-R. Lu, T.-S. Chan, E. Cortés, A. Fratalocchi, M. Liu, *Nano Energy* **2021**, 82, 105767.

[7] X. Zhang, L. Xia, G. Zhao, B. Zhang, Y. Chen, J. Chen, M. Gao, Y. Jiang, Y. Liu, H. Pan, W. Sun, *Advanced Materials* **2023**, 35, 2208821.

[8] L. Li, H. Qiu, Y. Zhu, G. Chen, S. She, X. Guo, H. Li, T. Liu, Z. Lin, H. Zhou, Y. Zhu, M. Yang, B. Xu, H. Huang, *Applied Catalysis B: Environmental* **2023**, 331, 122710.

[9] C. Yang, Z. Wu, Z. Zhao, Y. Gao, T. Ma, C. He, C. Wu, X. Liu, X. Luo, S. Li, C. Cheng, C. Zhao, *Small* **2023**, 19, 2206949.

[10] J. Li, Y. Li, J. Wang, C. Zhang, H. Ma, C. Zhu, D. Fan, Z. Guo, M. Xu, Y. Wang, H. Ma, *Advanced Functional Materials* **2022**, 32, 2109439.

[11] Q. He, D. Tian, H. Jiang, D. Cao, S. Wei, D. Liu, P. Song, Y. Lin, L. Song, *Advanced Materials* **2020**, 32, 1906972.

[12] Y. Zhu, M. Klingenhof, C. Gao, T. Koketsu, G. Weiser, Y. Pi, S. Liu, L. Sui, J. Hou, J. Li, H. Jiang, L. Xu, W.-H. Huang, C.-W. Pao, M. Yang, Z. Hu, P. Strasser, J. Ma, *Nature Communications* **2024**, 15, 1447.

[13] C. Li, S. H. Kim, H. Y. Lim, Q. Sun, Y. Jiang, H.-J. Noh, S.-J. Kim, J. Baek, S. K. Kwak, J.-B. Baek, *Advanced Materials* **2023**, 35, 2301369.

[14] Y. Dang, T. Wu, H. Tan, J. Wang, C. Cui, P. Kerns, W. Zhao, L. Posada, L. Wen, S. L. Suib, *Energy & Environmental Science* **2021**, 14, 5433.

[15] D. Chen, R. Yu, D. Wu, H. Zhao, P. Wang, J. Zhu, P. Ji, Z. Pu, L. Chen, J. Yu, S. Mu, *Nano Energy* **2022**, 100, 107445.

[16] M. Huang, H. Yang, X. Xia, C. Peng, *Applied Catalysis B: Environment and Energy* **2024**, 358, 124422.

[17] Y. Zhao, X. Wang, G. Cheng, W. Luo, *ACS Catalysis* **2020**, 10, 11751.

[18] G. Meng, H. Tian, L. Peng, Z. Ma, Y. Chen, C. Chen, Z. Chang, X. Cui, J. Shi, *Nano Energy* **2021**, 80, 105531.

[19] C. Li, B. Kim, Z. Li, R. Thapa, Y. Zhang, J.-M. Seo, R. Guan, F. Tang, J.-H. Baek, Y. H. Kim, J.-P. Jeon, N. Park, J.-B. Baek, *Advanced Materials* **2024**, 36, 2403151.

[20] Q. Hu, K. Gao, X. Wang, H. Zheng, J. Cao, L. Mi, Q. Huo, H. Yang, J. Liu, C. He, *Nature Communications* **2022**, 13, 3958.

[21] D. Yang, J.-H. Yang, Y.-P. Yang, Z.-Y. Liu, *Applied Catalysis B: Environmental* **2023**, 326, 122402.

[22] K. Jiang, M. Luo, Z. Liu, M. Peng, D. Chen, Y.-R. Lu, T.-S. Chan, F. M. F. de Groot, Y. Tan, *Nature Communications* **2021**, 12, 1687.

[23] J. Jiao, N.-N. Zhang, C. Zhang, N. Sun, Y. Pan, C. Chen, J. Li, M. Tan, R. Cui, Z. Shi, J. Zhang, H. Xiao, T. Lu, *Advanced Science* **2022**, 9, 2200010.

[24] H. Du, Z. Du, T. Wang, B. Li, S. He, K. Wang, L. Xie, W. Ai, W. Huang, *Advanced Materials* **2022**, 34, 2204624.

[25] Y. Wan, W. Chen, S. Wu, S. Gao, F. Xiong, W. Guo, L. Feng, K. Cai, L. Zheng, Y. Wang, R. Zhong, R. Zou, *Advanced Materials* **2024**, 36, 2308798.

[26] Y. Hong, S. Jeong, J. H. Seol, T. Kim, S. C. Cho, T. K. Lee, C. Yang, H. Baik, H. S. Park, E. Lee, S. J. Yoo, S. U. Lee, K. Lee, *Advanced Energy Materials* **2024**, 14, 2401426.

[27] H. Huang, H. Jung, S. Li, S. Kim, J. W. Han, J. Lee, *Nano Energy* **2022**, 92, 106763.

[28] D. Wang, W. Liu, H. Wang, S. Lu, Y. Li, S. Guo, Y. Xiang, *Advanced Functional Materials*, n/a, 2417976.

[29] L. Li, S. Liu, C. Zhan, Y. Wen, Z. Sun, J. Han, T.-S. Chan, Q. Zhang, Z. Hu, X. Huang, *Energy & Environmental Science* **2023**, 16, 157.

[30] J. Yin, T. Lu, J. Li, J. Liu, Y. Lin, D. Sun, L. Xu, Q. Zhao, H. Pang, S. Zhang, Y. Tang, *Advanced Functional Materials*, n/a, 2417034.

[31] D. S. Baek, H. Y. Lim, J. Kim, J. Lee, J. S. Lim, D. Kim, J. H. Lee, J.-W. Jang, S. K. Kwak, S. H. Joo, *ACS Catalysis* **2023**, 13, 13638.

[32] Z. Tao, H. Zhao, N. Lv, X. Luo, J. Yu, X. Tan, S. Mu, *Advanced Functional Materials* **2024**, 34, 2312987.

[33] B. Sarkar, D. Das, K. K. Nanda, *Journal of Materials Chemistry A* **2021**, 9, 13958.
